# Pan‐cancer analysis of genome‐wide methylation profiling discover type‐specific markers targeting circulating free DNA for the detection of colorectal cancer

**DOI:** 10.1002/ctm2.1370

**Published:** 2023-08-30

**Authors:** Lei Zhang, Dapeng Li, Lijing Gao, Ding Zhang, Qingzhen Fu, Hongru Sun, Shiheng Tan, Hao Huang, Ting Zheng, Tian Tian, Chenyang Jia, Haibo Zhou, Zinan Li, Lin Zhu, Xianyu Zhang, Da Pang, Shidong Xu, Lihong Hu, Weiwei Bao, Ning Zhao, Depei Zhang, Zesong Cheng, Yanlong Liu, Fan Wang, Binbin Cui, Yashuang Zhao

**Affiliations:** ^1^ Department of Epidemiology School of Public Health Harbin Medical University Harbin China; ^2^ Department of Breast Surgery Tumor Hospital of Harbin Medical University Harbin Medical University Harbin China; ^3^ Department of Chest Surgery Tumor Hospital of Harbin Medical University Harbin Medical University Harbin China; ^4^ Department of Gastroenterology The Second Affiliated Hospital of Harbin Medical University Harbin Medical University Harbin China; ^5^ Department of Colorectal Surgery Tumor Hospital of Harbin Medical University Harbin Medical University Harbin China; ^6^ NHC Key Laboratory of Etiology and Epidemiology Harbin Medical University Harbin China

Dear Editor,

Colorectal cancer (CRC) is one of the most commonly diagnosed cancers and cancer‐related causes of death worldwide.^1^ Early diagnosis is critical to provide curable treatment and improving survival rates for CRC patients.^2^ Circulating‐free DNA (cfDNA) carrying cancer‐specific methylation signature is a promising specific marker for cancer diagnosis.^3^ However, previous studies were based either on high‐throughput sequencing or did not consider the specificity of the methylation pattern for different cancers. We aim to discover and validate a noninvasive method with type‐specific DNA methylation patterns for the diagnosis of CRC.

The workflow is illustrated in Figure 1 (details of the sample sources and analysis process can be found in the Supporting information). Briefly, we first compared the differentially methylated CpG sites (DMCs) between 395 CRC and 45 adjacent normal tissues from the cancer genome atlas (TCGA) dataset, and a total of 37,132 DMCs were selected based on |Δβ| > 0.20 and FDR < 0.05. We further filtered out 27,063 CpG sites due to potential noise of DNA methylation (average beta > 0.1 or < 0.9) in 1,246 samples of white blood cells (WBCs) of healthy individuals from two Gene Expression Omnibus (GEO) datasets. Finally, with the same filtering criteria as WBCs, 15 CRC‐specifically hypermethylated CpG sites with average methylation levels less than 0.1 in 8,629 tissue samples from 28 other cancer types in the TCGA dataset were retained, and no CRC‐specific hypomethylated CpG sites met the criteria and were retained. The heatmap showed that the 15 CpG sites (located on *B3GALNT1*, *C6orf97*, *FAM72A*, *FAM72B*, *LIFR*, *OSMR*, *ZNF264*, and *ZNF543*) well‐distinguished CRC from adjacent normal tissues (Figure 2A), WBCs (Figure 2B), and 28 other types of cancer (Figure 2C) in TCGA, as well as 23 other types of cancer in GEO (Supporting information). In addition, DNA methylation has a potential role in regulating gene expression, and CpG sites, including cg14786398, were negatively correlated with their corresponding gene expression (*r* = −0.691, *p* < 0.001, Figure 2D, Supporting information).

**FIGURE 1:**
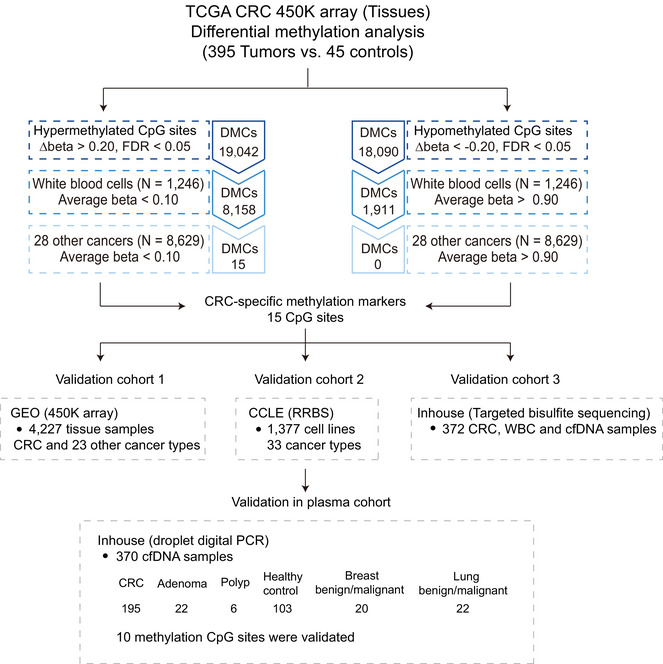
A schematic of the study design. TCGA: the cancer genome atlas; CRC: colorectal cancer; FDR: false discovery rate; DMCs: methylated CpG sites; WBC: white blood cell; RRBS: reduced representation bisulfite sequencing.

**FIGURE 2:**
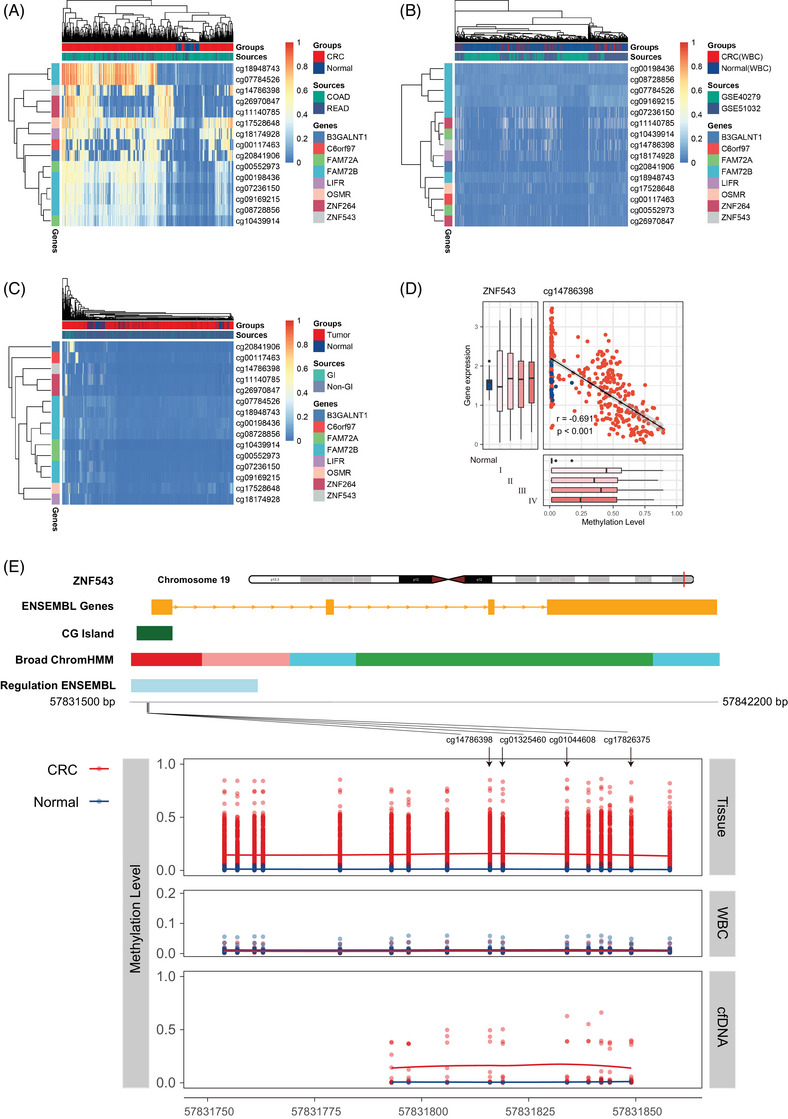
Methylation levels of colorectal cancer‐specific CpG sites in the DNA of colorectal cancer tissues, adjacent normal tissues, white blood cells, other types of tumor tissues and plasma cfDNA. (A) Unsupervised hierarchical clustering of the 15 colorectal cancer (CRC)‐specific DNA methylation markers in 395 CRC samples and 45 matched adjacent normal tissue samples from the TCGA database. (B) Unsupervised hierarchical clustering of 15 CRC‐specific DNA methylation markers in white blood cells (WBCs) of 166 CRC patients (at the last follow‐up) and 1 080 healthy individuals from the GEO database. (C) Unsupervised hierarchical clustering of 15 CRC‐specific DNA methylation markers in 7,925 tumour and 704 normal tissues of 28 other cancer types from the TCGA database (Table S1). Gastrointestinal (GI)‐related tumours mainly include esophageal carcinoma (ESCA), hepatocellular carcinoma (LIHC), pancreatic adenocarcinoma (PAAD) and stomach adenocarcinoma (STAD). (D) Spearman correlation analysis between methylation levels cg14786398 and gene expression of *ZNF543* in the TCGA CRC dataset. The boxplots on the left and below represent the level of gene expression and methylation of CpG in CRC tissues at different tumor stages and adjacent normal tissues, respectively. (E) Gene model of *ZNF543* and the methylation level of the *ZNF543* target region in the validation cohort. The top panel shows the methylation level of 227 CRC tissues and 24 adjacent normal tissues, where each dot represents one CpG site for each sample. The middle panel shows the methylation level of white blood cells (WBCs) from CRC patients (*n* = 27) and healthy controls (*n* = 25). The lowest panel shows the methylation level of cfDNA samples from CRC patients (*n* = 9) and healthy controls (*n* = 5).

The area under curve (AUC) of the 15 CRC‐specific CpG sites ranged from 0.643 to 0.903, similar to the frequently reported CpG sites of *SEPT9* (0.846 to 0.967). Compared with the *SEPT9*, our markers have higher CRC specificity, and the misclassification rate of the 15 CpG sites in 28 other types of tumor tissues ranged from 0% to 20%, while the misclassification rates of 11 CpG sites in *SEPT9* ranged from 0% to 92% (Supporting information).

Furthermore, the methylation status of selected CRC‐specific markers was evaluated by MethylTarget sequencing (Genesky) in CRC tissue (*N* = 227), adjacent normal tissue (*N* = 24), WBC (*N* = 52) and cfDNA (*N* = 14) samples from CRC patients and healthy controls. The candidate CpG sites of *ZNF543* were significantly hypermethylated in tissues and cfDNA from CRC compared to normal tissues and cfDNA from healthy controls and were unmethylated in WBCs from both CRC and healthy controls (Figure 2E). Candidate CpG sites of *B3GALNT1*, *C6orf97*, *LIFR*, and *ZNF264* were also hypermethylated in CRC tissues but unmethylated in normal tissues and WBCs (Supporting information).

We successfully designed primers and probes for six CRC‐specific markers (*FAM72A*, *FAM72B*, *LIFR*, *OSMR*, *ZNF264* and *ZNF543*) covering 10 of 15 CpGs for further testing the methylation level of cfDNA with ddPCR‐based assays (Figure 3A; Supporting information). The established three multiplex ddPCR assays (mddPCR, Assay 1, 2, 3) have superior detection sensitivity compared with traditional multiplex MethyLight (mqPCR). mqPCR assay 1 detected one methylated allele in a background of 125 unmethylated alleles (Figure 3B; limit of quantification (LOQ) = 0.8%, *R* ^2^ = 0.957). In contrast, the LOQ of Assay 1 was 25‐fold lower than that of mqPCR assay 1 (Figure 3C,D, Supporting information).

**FIGURE 3:**
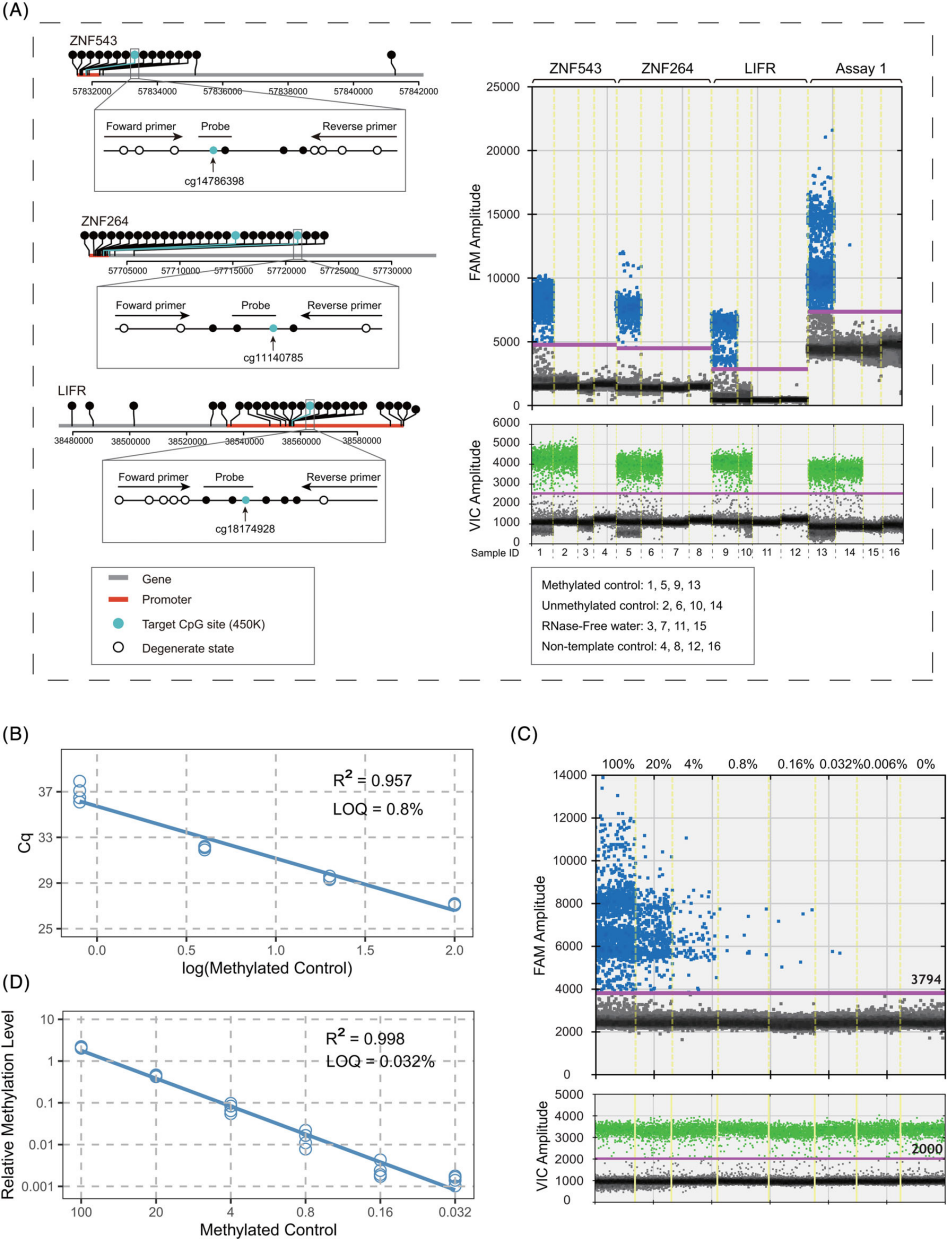
Comparative analysis of the limit of quantification (LOQ) for mddPCR Assay 1 with conventional multiplex MethyLight PCR (mqPCR). (A) Schematic illustration of the localization of target CpG sites (Infinium 450K) and primers and probes for droplet digital PCR assays related to the presence of CpG sites in the genomic region of *ZNF543*, *ZNF264*, and *LIFR* (left panel). Combination and construction of Assay 1 (right panel). The solid pink line is the manually set threshold for dividing positive and negative droplets. (B) Standard curve of quantification between Cq value and log transformation of serially diluted methylated controls in mqPCR assay 1. (C) Quantasoft amplification plots of a 5‐fold dilution series of methylated controls in Assay 1. The solid pink line is the manually set threshold, which is used for dividing positive and negative droplets. (D) Standard curve of quantification between relative methylation level and serially diluted methylated controls in Assay 1. All DNA controls were run in four replicates. The *x*‐axis displays the concentration (or log‐transformed concentration) of methylated controls, and the *Y*‐axis represents the values of the quantitative cycle (Cq) or relative methylation level (the ratio of methylated molecule copies of the target region to that of the reference gene (ACTB) of each methylated control).

A total of 370 blood samples were collected from 195 patients with CRC, 6 patients with hyperplastic polyps, 22 patients with advanced adenomas (AAs), 103 healthy controls, and 44 non‐CRC patients with benign or malignant tumours of breast or lung. The AUCs of the three mddPCR assays for distinguishing CRC patients from healthy controls were 0.767, 0.847 and 0.771 (Figure 4A–C), respectively. Samples with detected methylated molecules were judged as positive, with a sensitivity of 57.9% for Assay 1, 71.8% for Assay 2 and 57.9% for Assay 3. The corresponding specificities were 92.2%, 94.2% and 95.1%, respectively. Furthermore, elevated methylated molecule copies were also detected in patients with AA, with positive rates of 31.8%, 22.7% and 31.8% of the three mddPCR assays. Next, we combined three mddPCR assays to evaluate the combined diagnostic performance. The AUCs of the four combination panels were 0.884, 0.821, 0.870 and 0.892, respectively (Figure 4D). Based on the optimal cutoff values, the sensitivities of the four panels were 81.5%, 70.3%, 77.9% and 84.1%, and the corresponding specificities were 89.3%, 88.3%, 90.3% and 85.4%, respectively (Figure 4D). Furthermore, the AUCs for diagnosis of non‐CRC were relatively low, ranging from 0.541 to 0.599.

**FIGURE 4:**
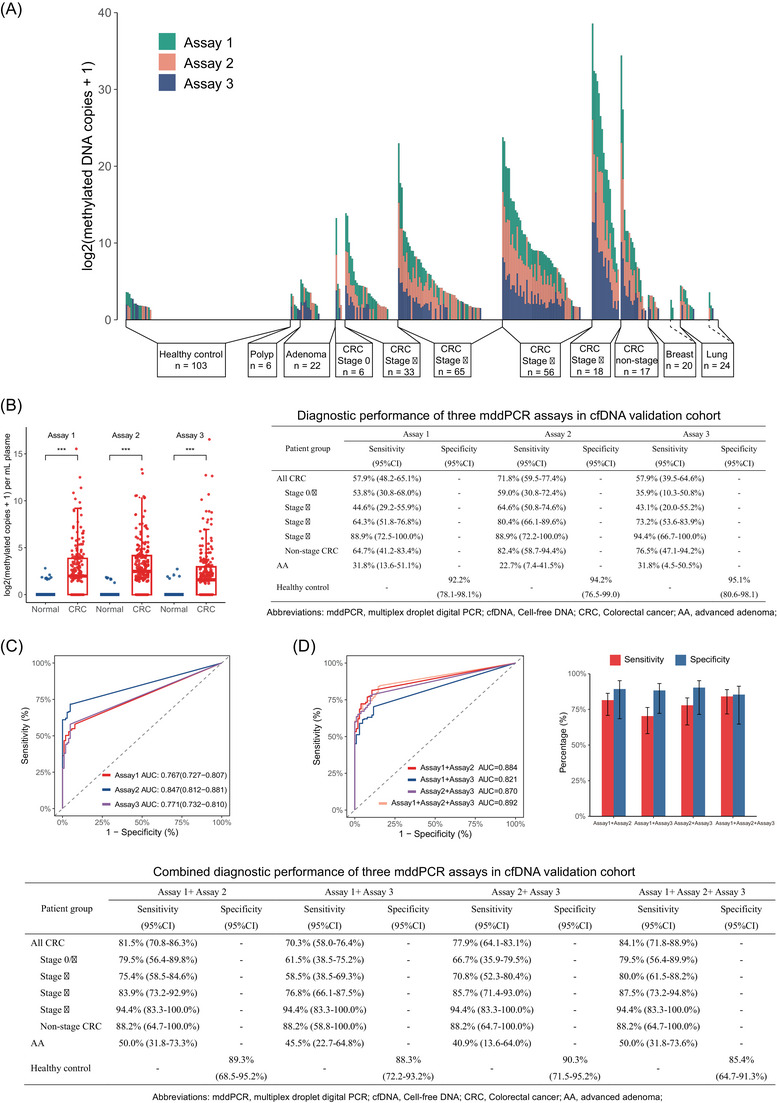
Diagnostic performance of three mddPCR assays in cfDNA. (A) The number of methylated DNA copies detected per mL of plasma in the three mddPCR assays and the sum. The *x*‐axis is the type and number of all samples, and each stacked bar represents a single participant. Patients with malignant and benign diseases of the breast and lung are separated by dotted lines. (B) Boxplot and dotplot of the number of methylated molecule copies in three mddPCR assays. ***: *P* ≤ 0.001. (C) Receiver operating characteristic (ROC) curves of Assay 1, Assay 2 and Assay 3 for distinguishing colorectal cancer (CRC) from healthy controls. (D) ROC curves, sensitivities and specificities of four combination panels for distinguishing CRC from healthy controls. Error bars represent 95% confidence interval (CI).

Identification of the origin of cfDNA and the location of the cancer is critical for guiding clinical diagnosis. Currently, *mSEPT9* is the only blood assay in the clinical setting for CRC screening, but its clinical usefulness is limited by its low sensitivity in early‐stage CRC.^4^ Moreover, *mSEPT9* is not a CRC‐specific marker because of overlapping aberrant methylation across multiple cancers.^5^, ^6^ Recent evidence suggests that the use of tissue‐specific methylation signatures will allow for tracing tissue of origin in cfDNA.^7^, ^8^ In marker discovery, we eliminated the possible confounding interference of cfDNA released by other cancer tissues or WBCs on the detection of ctDNA methylation levels in CRC. Moreover, our inhouse validation study suggested that such CRC‐specific methylation patterns could be detected in tissues and cfDNA but not in WBCs.

This study has several limitations. First, most of the healthy controls and non‐CRC patients of our inhouse cfDNA cohort were not confirmed by colorectal endoscopy, thus, those positive results were classified as false‐positives to more closely reflect the situation in the natural population in the real world, and this could have resulted in an underestimation of diagnostic performance of our mddPCR assays to a certain extent. Second, we collected a small subset of cfDNA samples from patients with AA and other non‐CRC diseases, suggesting that our arrays need to be further optimized and validated in studies including more participants in the future.

In conclusion, we identified a panel of CRC‐specific methylation patterns by pan‐cancer analysis and developed three cfDNA multiplex ddPCR assays. Our findings suggested that cfDNA methylation assays have the potential to detect early‐stage CRC and its advanced precursors. However, the diagnostic performance of these arrays requires more validation before clinical implementation.

## CONFLICT OF INTEREST STATEMENT

The authors declare no conflicts of interest.

## Supporting information

Supporting information
