# Supplementary material for: Pan‐cancer analysis of genome‐wide methylation profiling discover type‐specific markers targeting circulating free DNA for the detection of colorectal cancer
Source: Clin Transl Med. 2023 Aug 30;13(9):e1370. doi: 10.1002/ctm2.1370 (PMC10468582; doi:10.1002/ctm2.1370)
Supplement: Supplementary file 1 — Supporting information [file CTM2-13-e1370-s001.docx]

**Supplementary information**

**Pan-cancer analysis of genome-wide methylation profiling discover type-specific markers targeting circulating free DNA for the detection of colorectal cancer**

Contents

[Supplementary Methods 4](#_Toc140762618)

[Source of public datasets 4](#_Toc140762619)

[Inhouse subject recruitment 5](#_Toc140762620)

[Sample collection and processing 6](#_Toc140762621)

[Selection of CRC tissue-specific methylation markers 6](#_Toc140762622)

[MethylTarget sequencing for samples of the inhouse primary validation cohort 7](#_Toc140762623)

[ddPCR methylation analysis for samples of the inhouse cfDNA validation cohort 8](#_Toc140762624)

[Statistical analysis 9](#_Toc140762625)

[Supplementary results 10](#_Toc140762626)

[Validation of CRC-specific methylation markers by NGS-based target sequencing 11](#_Toc140762627)

[Development of cfDNA multiplex ddPCR assays 12](#_Toc140762628)

[The detective sensitivity of cfDNA methylation array 12](#_Toc140762629)

[Diagnostic performance of cfDNA arrays in AA and CRC 13](#_Toc140762630)

[Table S1. Public and inhouse datasets used in this study. 17](#_Toc140762631)

[Table S2. Primer sequences of candidate CRC-specific methylation markers for targeted bisulfite sequencing. 20](#_Toc140762632)

[Table S3. Primer and probe sequences of candidate CRC-specific methylation markers and reference gene in ddPCR. 21](#_Toc140762633)

[Table S4. Composition of the three multiplex droplet digital PCR assays. 22](#_Toc140762634)

[Table S5. The genomic characteristics of 15 CRC-specific methylation CpG sites. 23](#_Toc140762635)

[Table S6. The genomic characteristics of 12 CpG sites of *SEPT9* island. 24](#_Toc140762636)

[Table S7. Diagnostic performance analyses of 15 CpG sites for distinguishing colorectal cancer from adjacent normal tissues in four datasets. 25](#_Toc140762637)

[Table S8. Diagnostic performance analyses of 11 CpG sites of *SEPT9* island for distinguishing colorectal cancer from adjacent normal tissues in four datasets. 26](#_Toc140762638)

[Table S9. Diagnostic performance analyses of 15 CpG sites for distinguishing adenoma from adjacent normal tissues in three datasets. 27](#_Toc140762639)

[Table S10. Receiver operating characteristic curve analyses of 16 CpG sites in target region of *B3GALNT1* between colorectal cancer and adjacent normal tissues in our inhouse primary validation dataset. 28](#_Toc140762640)

[Table S11. Receiver operating characteristic curve analyses of 31 CpG sites in target region of *C6orf97* between colorectal cancer and adjacent normal tissues in our inhouse primary validation dataset. 29](#_Toc140762641)

[Table S12. Receiver operating characteristic curve analyses of 17 CpG sites in target region of *LIFR* between colorectal cancer and adjacent normal tissues in our inhouse primary validation dataset. 30](#_Toc140762642)

[Table S13. Receiver operating characteristic curve analyses of 36 CpG sites in target region of *ZNF264* between colorectal cancer and adjacent normal tissues in our inhouse primary validation dataset. 31](#_Toc140762643)

[Table S14. Receiver operating characteristic curve analyses of 16 CpG sites in target region of *ZNF543* between colorectal cancer and adjacent normal tissues in our inhouse primary validation dataset. 32](#_Toc140762644)

[Table S15. The demographic and clinical characteristics of the CRC, precancerous lesions, and healthy controls in the cfDNA validation cohort. 33](#_Toc140762645)

[Table S16. The demographic and clinical characteristics of the CRC and healthy controls in the age-matched cfDNA validation cohort. 34](#_Toc140762646)

[Figure S1. Validation of 15 CRC-specific methylation CpG sites in multiple types of cancer tissues and normal tissues from GEO database. 35](#_Toc140762647)

[Figure S2. Methylation levels of 11 CpG sites in the *SEPT9* promoter in 29 types of cancer tissues from TCGA database. 36](#_Toc140762648)

[Figure S3. Validation of 11 CpG sites in the *SEPT9* promoter in multiple types of cancer tissues and normal tissues from GEO database. 37](#_Toc140762649)

[Figure S4. Heatmap for misclassification rate of our 15 CpG sites and 11 CpG sites of *SPET9* for distinguishing CRC samples from 28 other cancer types in TCGA dataset. 38](#_Toc140762650)

[Figure S5. Boxplots for eight gene expression in CRC tissues and adjacent normal tissues. 39](#_Toc140762651)

[Figure S6. Spearman correlation analysis between methylation levels of nine CpG sites and gene expression of *FAM72A*, *C6orf97*, and *FAM72B* in the TCGA CRC dataset. 40](#_Toc140762652)

[Figure S7. Spearman correlation analysis between methylation levels of six CpG sites and gene expression of *B3GALNT1*, *LIFR*, *OSMR* and *ZNF264* in the TCGA CRC dataset. 41](#_Toc140762653)

[Figure S8. Methylation (RRBS) level (A) and expression level (B) of *B3GALNT1* gene in multiple types of cancer cell lines from Cancer Cell Line Encyclopedia. (C) Methylation (RRBS) of *FAM72B*. (D) mRNA expression (RNA-seq) of *FAM72B*. 42](#_Toc140762654)

[Figure S9. Methylation (RRBS) level (A) and expression level (B) of *LIFR* gene in multiple types of cancer cell lines from Cancer Cell Line Encyclopedia. (C) Methylation (RRBS) of *OSMR*. (D) mRNA expression (RNA-seq) of *OSMR*. 43](#_Toc140762655)

[Figure S10. Methylation (RRBS) level (A) and expression level (B) of *ZNF264* gene in multiple types of cancer cell lines from Cancer Cell Line Encyclopedia. (C) Methylation (RRBS) of *ZNF543*. (D) mRNA expression (RNA-seq) of *ZNF543*. 44](#_Toc140762656)

[Figure S11. Methylation (RRBS) level (A) and expression level (B) of *FAM72A* gene in multiple types of cancer cell lines from Cancer Cell Line Encyclopedia. (C) Methylation (RRBS) of *C6orf97*. (D) mRNA expression (RNA-seq) of *C6orf97*. 45](#_Toc140762657)

[Figure S12. Correlation between methylation and expression levels of (A) *B3GALNT1*, (B) *FAM72B*, (C) *LIFR*, (D) *OSMR*, (E) *ZNF264*, (F) *ZNF543*, (G) *FAM72A* and (H) *C6orf97* in CRC cell lines. 46](#_Toc140762658)

[Figure S13. Validation of 15 CRC-specific methylation CpG sites of our study (A) and 11 CpG sites in the *SEPT9* promoter (B) in tissues of colorectal adenomas and normal tissue samples. 47](#_Toc140762659)

[Figure S14. The methylation level of the target region in the validation cohort. 48](#_Toc140762660)

[Figure S15. Schematic illustration of the localization of target CpG sites (Infinium 450K) and droplet digital PCR assays related to the presence of CpG sites in the genomic region of *FAM72A* (A), *FAM72B* (B), and *OSMR* (C). 49](#_Toc140762661)

[Figure S16. Combination and construction of multiplex droplet digital PCR assays. (A) Assay 2, and (B) Assay 3. 50](#_Toc140762662)

[Figure S17. Comparative analysis of limit of quantification (LOQ) for multiplex droplet digital PCR (mddPCR) and conventional multiplex MethyLight PCR (mqPCR). 51](#_Toc140762663)

[Figure S18. Analysis of cfDNA extraction concentrations in our cfDNA validation cohort. 52](#_Toc140762664)

[Figure S19. Receiver operating characteristics curves of Assay 1, Assay 2, and Assay 3 for distinguishing colorectal cancer from healthy controls in original or age-matched cfDNA cohort. 53](#_Toc140762665)

# Supplementary Methods

## Source of public datasets

DNA methylation data of more than 480,000 CpG sites generated using Infinium 450K methylation array were obtained from The Cancer Genome Atlas (TCGA) and Gene Expression Omnibus (GEO). The methylation level of the CpG sites range from 0 to 1 (called β value), which is a ratio of intensities between methylated (M) allele and the sum of methylated (M) and unmethylated (U) allele. Illumina 450K methylation array of tissue samples from 29 different types of cancer were downloaded from UCSC Xena (https://xena.ucsc.edu/public/), including 395 colorectal cancer (CRC) tissues, 45 matched adjacent normal tissues, and 8,629 tissues of 28 other cancer types. Additionally, 35 methylation datasets were obtained from GEO, involving 1,246 samples of white blood cells (WBCs) from healthy individuals (GSE40279 and GSE51032), 604 samples of tissues from CRC patients (GSE48684 ^1^, GSE101764 ^2^, and GSE131013 ^3^), 166 samples of tissues from adenoma patients (GSE48684 ^1^, GSE77954 ^4^ and GSE139404 ^5^), and 3,457 samples of tissues from twenty-three other cancer types (GSE77871 ^6^, GSE111933 ^7^, GSE66695 ^8^, GSE69914 ^9^, GSE68339 ^10^, GSE49656 ^11^, GSE178212 ^12^, GSE79366 ^13^, GSE60274 ^14^, GSE36278 ^15^, GSE79556 ^16^, GSE61441 ^17^, GSE157341 ^18^, GSE39279 ^19^, GSE155353 ^20^, GSE49149 ^21^, GSE43293 ^22^, GSE112047 ^23^, GSE40853 ^24^, GSE120878 ^25^, GSE85464 ^26^, GSE164988 ^27^, GSE74104 ^28^, GSE97466 ^29^, GSE86961 ^30^, GSE94769 ^31^, GSE67116 ^32^ and GSE136790 ^33^). Furthermore, Reduced Representation Bisulfite Sequencing (RRBS) and gene expression parallel sequencing data from 1,377 cancer cell lines for 33 cancer types were obtained from the Cancer Cell Line Encyclopedia (CCLE, http://www.broadinstitute.org/ccle/home). Details of the included subjects are presented in Table S1.

## Inhouse subject recruitment

All inhouse subjects were recruited at the Second Affiliated Hospital or Tumor Hospital of Harbin Medical University. For the inhouse primary validation cohort, 300 colorectal cancer tissue samples, 58 white blood cell samples and 14 cfDNA samples were collected. For the inhouse cfDNA validation cohort, 370 cfDNA samples of colorectal cancer, precancerous lesions, non-CRC patients (benign or malignant tumors of breast or lung) and healthy controls were collected. Tissue samples were collected between November 2004 and January 2008, and WBC samples were collected from October 2017 to July 2018. Participants who were ever diagnosed with other malignancies were excluded. Patients with neuroendocrine carcinoma, malignant melanoma, non-Hodgkin's lymphoma, gastrointestinal stromal tumor, or Lynch syndrome in the gastrointestinal tract were excluded. All subjects were newly diagnosed by postoperative pathology and underwent surgery without chemotherapy or radiotherapy. Blood samples of patients and healthy controls were collected from February 2020 to December 2021. Healthy controls were recruited from volunteers who were free from history of malignancy, and no CRC-related symptoms within 30 days prior to enrollment. Demographic and clinical information of the study subjects was obtained from medical records and questionnaires. Informed consent was obtained from all subjects. The study protocol was approved by the Medical Ethics Committee of Harbin Medical University. Details of the sample sources and sample sizes can be found in Table S1.

## Sample collection and processing

The tissue samples were immediately snap-frozen in liquid nitrogen and stored at -80 ℃ until used for analysis. For cfDNA, 8-10 mL of whole blood samples were collected prior to any treatment and plasma was separated within 4 h using Ardent Cell-Free DNA blood collection tubes or within 2 h using EDTA K2 tubes. Whole blood samples were centrifuged at 3,000 rpm for 10 minutes at room temperature, then the plasma supernatant was carefully collected and centrifuged again at 16,000g for 10 min at 4℃. Plasma can be used immediately for cfDNA extraction or frozen at -80°C. During cfDNA extraction, carrier RNA was added as recommended in the QIAamp Circulating Nucleic Acid kit (Qiagen) kit instruction to reduce the loss of free nucleic acid. Chen et al ^34^ and our previous study results suggested that carrier RNA may affect the quantification of cfDNA by Qubit 3.0 Fluorometer, but the bias was the same for all cfDNA samples and does not interfere with methylation detection. The extracted DNA samples and control DNAs were bisulfite converted using the EZ DNA Methylation-Gold™ Kit (Zymo Research). All extractions or bisulfite conversion of DNA were performed according to the manufacturer’s guidelines.

## Selection of CRC tissue-specific methylation markers

The filtering process of CRC-specific methylation markers is shown in Figure 1. First, CpG sites with more than 10% missing values in 440 CRC samples were removed, and the remaining were imputed using the ‘impute’ package (version 1.64.0). Probes affected by common SNPs or located on the X and Y chromosomes were removed after gene annotation of the imputed CpG sites (‘IlluminaHumanMethylation450kanno.ilmn12.hg19’, version 0.6.0). Differential methylation analysis of imputed CpG sites between CRC (N = 395) and normal tissues (N = 45) was performed using the ‘DMRcate’ package (version 2.4.1) and adjusted by the Benjamini-Hochberg method to obtain false discovery rate (FDR). Differentially methylated CpG sites (DMCs) with FDR less than 0.05 (FDR < 0.05) and absolute methylation differences between the two groups greater than 0.2 (|Δβ| > 0.20) were retained (n = 37,132). Second, methylation levels of DMCs were assessed in 1,246 sample of white blood cells (WBCs) from healthy individuals. For differentially hypermethylated CpG sites, remove CpG sites with an average methylation level greater than 0.1 in WBCs; for differentially hypomethylated CpG sites, remove CpG sites with an average methylation level less than 0.9 in WBCs. Finally, the remaining 10,069 DMCs were further assessed for methylation levels in 8,629 tissue samples from 28 other cancer types, The filtering criteria are the same as WBCs. For differentially hypermethylated CpG sites and differentially hypomethylated CpG sites, CpG sites with average methylation levels greater than 0.1 or less than 0.9 in other types of tumor tissues are excluded, respectively.

At last, 15 CRC hypermethylated CpG sites with average methylation levels less than 0.1 in tissues of 28 other cancer types were retained, and no CRC-specific hypomethylated CpG sites met the criteria and were retained.

## MethylTarget sequencing for samples of the inhouse primary validation cohort

MethylTarget sequencing (Genesky), which is an NGS-based targeted bisulfite sequencing method, was performed on samples of our inhouse primary validation cohort. PCR primers were designed with methylation primer software (primer3) based on the bisulfate converted DNA sequence. Primers were designed to cover 100-250 nucleotides adjacent to the candidate CpG sites instead of directly covering the candidate CpG sites. The sequences of the primers are listed in Table S2. A two-step PCR approach was performed for each bisulfite-converted DNA sample, with multiplexed PCR amplification followed by index PCR. The enriched libraries were sequenced using the Illumina HiSeq platform with a 2x150 bp paired-end mode. After quality control and filtering, paired reads were merged and mapped to the reference genome of human GRCh37/hg19. The methylation level of each CpG site was calculated as methylated read counts divided by total read counts. Samples with a bisulfite conversion rate < 98% or average coverage less than ×10 were filtered out.

## ddPCR methylation analysis for samples of the inhouse cfDNA validation cohort

Sensitive and specific TaqMan probes were designed for cfDNA methylation detection. Primers and probes were designed using ABI Primer Express software (version 3.0.1) or manually. Primer and probe sequences of the ddPCR assays are listed in Table S3. All primers and probes were synthesized by Sangon Biotech (Shanghai, China). cfDNA methylation analysis was performed on the QX200™ Droplet Digital™ PCR System (Bio-Rad). The total volume of the PCR mixture was 21 μL, containing 10 μL of ddPCR™ Supermix for Probes (No dUTP), adjusted volumes of primers and probes, and 7-8 μL template DNA (Table S4). The heat-sealed plates were placed in a T100 Thermal cycler and amplified for 40 cycles to the endpoint. The PCR conditions were as follows: 10 min at 95 °C, 40 cycles of 94 °C for 30 s and 60 °C for 1 min; 10 min hold at 98 °C. The ramp rate was set at 2 ℃/s for all steps.

Four control samples were included in each ddPCR plate: a methylated DNA control, an unmethylated DNA control, an RNase-free water control, and a non-template control (NTC). Data from the QX200 Droplet Reader were analyzed in QuantaSoft version 1.7.4 (Bio-Rad). The fluorescence thresholds for calling droplets positive or negative were set manually based on the distribution of droplets of control samples. The same threshold was applied to all the wells of one PCR plate. Samples with a total number of droplets in a single well less than 8000 or no positive droplets in the VIC channel were excluded. In the FAM channel, samples with at least two droplets with fluorescent amplitudes above the threshold were considered positive, and for those positive samples, the copies of methylated alleles per mL of plasma were calculated.

## Statistical analysis

Comparisons between groups were determined by analysis of variance, t test, Mann-Whitney U test, Chi-square test, or Fisher's exact test, as appropriate. Correlation analysis was conducted with the Pearson or Spearman method. The optimal cutoff value of each marker was determined using the receiver operating characteristic (ROC) curve at the maximal Youden index. The misclassification rate of predicting CRC in non-CRC tumor and non-CRC normal samples from 28 other cancer types was calculated at the same cutoff value. The diagnostic performance of individual markers was evaluated by sensitivity, specificity, and area under the ROC curve (AUC). Under the null hypothesis of AUC = 0.75, a sample of 105 for each group would ensure a two-sided test with α = 0.05 and 90% power to detect a 10% difference ^35^. All statistical analyses were performed with R software (version 4.0.4). A two-sided *P* value less than 0.05 was considered significant. The genome reference version used in this study was GRCh37/hg19.

# Supplementary results

The workflow and subject enrollment in this study are illustrated in Figure 1 and Table S1. Briefly, we identified 15 CRC-specifically hypermethylated CpG sites by pan-cancer analysis (Table S5). The heatmap showed that the 15 CpG sites well distinguished CRC from adjacent normal tissues (Figure 2A), WBCs (Figure 2B) and 28 other types of cancer (Figure 2C) in TCGA, as well as CRC-tissue specific hypermethylation in 24 cancer types from GEO (N = 4,061, Figure S1). In addition, we collected frequently reported CpG sites in *SEPT9* (Table S6). In contrast, these CpG sites showed aberrant hypermethylation not only in patients with CRC, but also in patients with Bladder Cancer (BLCA), Head and Neck Cancer (HNSC), liver cancer (LIHC), and stomach cancer (STAD, Figure S2, Figure S3). The misclassification rate of the 15 CpG sites ranged from 0 to 20% in the other 28 types of cancer tissues, while the misclassification rates of the 11 CpG sites of *SEPT9* ranged from 0 to 92% (Figure S4).

Aberrant DNA hypermethylation is often involved in regulating gene downregulation or silencing. The expression levels of *LIFR* and *ZNF264* were downregulated in CRC tissues compared with that of adjacent normal tissue (Figure S5). The methylation levels of cg07784526, cg18948743, cg08728856, cg00198436, cg07236150 and cg09169215 on *FAM72B* gene were negatively correlated with gene expression, and correlation coefficients range from -0.458 to -0.729 (Figure S6). The correlation between methylation levels and gene expression of cg26970847 and cg11140785 located on the *ZNF264* gene was -0.635 and -0.672, respectively (Figure S7). The correlation between the methylation level of cg20841906, cg18174928 and cg17528648 and the expression of *B3GALNT1*, *LIFR* and *OSMR* were -0.407, -0.346 and -0.414, respectively (Figure S7).

In addition, data from various cancer cell lines also showed that these genes exhibit specific hypermethylation in CRC, accompanied by low expression (N = 1,377, Figure S8-S11). Similar to the CRC tissue samples, the promoter methylation levels of *B3GALNT1*, *FAM72B*, *OSMR* and *ZNF264* were negatively correlated with the corresponding gene expression levels in colorectal cancer cell lines (Figure S12).

Analyzing the performance for CRC, the AUCs of the 15 CRC-specific CpG sites of our study and the 11 CpG sites in the *SEPT9* promoter ranged from 0.643 to 0.903 (Table S7) and 0.846 to 0.967 (Table S8), respectively. In addition, these 15 CRC-specific CpG sites can identify a significant proportion of adenomas (Figure S13), with AUCs ranging from 0.647 to 0.879 (Table S9).

## Validation of CRC-specific methylation markers by NGS-based target sequencing

Samples that did not pass quality control were excluded, including 3 cases with bisulfite conversion rate < 98% and 55 cases with average coverage less than ×10. We examined the methylation status of the selected genes in our inhouse primary validation cohort with CRC tissue (N = 227), adjacent normal tissue (N = 24), WBC (N = 52) and cfDNA (N = 14) samples from CRC patients and healthy controls. In addition to the example gene *ZNF543* (Figure 2E), candidate CpG sites located on *B3GALNT1*, *C6orf97*, *LIFR* and *ZNF264* were also hypermethylated in CRC tissues but unmethylated in normal tissues and WBCs (Figure S14). These targeted CpG sites had similar diagnostic performance as the discovery cohort in distinguishing CRC from adjacent normal tissues (AUCs ranged from 0.646 to 0.823, Table S10-S14).

## Development of cfDNA multiplex ddPCR assays

Eight droplet digital PCR assays were successfully designed (Table S3, Figure 3A, Figure S15). We developed the multiplex ddPCR (mddPCR) assay with two different fluorescences. The FAM reporter was used to label the methylated probes of candidate CpG sites, and the VIC reporter was used to label the reference gene (ACTB). According to the background amplitude of each amplified fragment (template is the methylated DNA control) and the fluorescence signal distribution of positive and negative droplets, seven single assays were integrated into three multiplex digital PCR assays as follows: Assay 1 (*LIFR*, *ZNF264*, and *ZNF543*), Assay 2 (*FAM72A* and *FAM72B*_2), and Assay 3 (*FAM72B*_1 and *OSMR*) (Table S4, Figure 3A, and Figure S16).

## The detective sensitivity of cfDNA methylation array

The methylated DNA control was mixed with the unmethylated DNA control at 100%, 20%, 4%, 0.8%, 0.16%, 0.032%, 0.006% and 0%, with a total DNA amount of approximately 10 ng per reaction. Dilution series of methylated controls were both run in mddPCR assays and MethyLight PCR assays (mqPCR). mqPCR assay 1 detected one methylated allele in a background of 125 unmethylated alleles (Figure 3B; limit of quantification (LOQ) = 0.8%, R^2^ = 0.957). In contrast, the FAM channel of Assay 1 showed that the number of positive droplets decreased with the serial dilution of standards until the concentration dropped to 0.032%, which means that Assay 1 could detect one methylated allele in a background of 3,125 unmethylated alleles (Figure 3C-D, R^2^ = 0.998). This was a 25-fold lower LOQ than that of mqPCR assay 1. The detection performance of Assay 2 and Assay 3 was similar to that of Assay 1 (Figure S17).

## Diagnostic performance of cfDNA arrays in AA and CRC

We recruited 195 patients with CRC, 6 patients with hyperplastic polyps, 22 patients with AAs, and 103 healthy controls. The detailed characteristics of the participants are shown in Table S15. CRC patients yielded a median cfDNA concentration of 7.98 ng/mL plasma (IQR: 6.21-10.31), whereas healthy controls had a median concentration of 6.90 ng/mL plasma (IQR: 4.96-9.37, *P* < 0.01, Figure S18). The numbers of methylated molecule copies showed a trend of stepwise increase with the progression of CRC (Figure 4A). In addition, three multiplex ddPCR assays demonstrated acceptable AUCs in distinguishing CRC from healthy controls and identified a subset of precancerous lesions (Figure 4B). As patients in the CRC group were older (median age = 60.3) than the healthy controls (median age = 51.3), we performed an age-matched analysis (N = 184, Table S16), and the three arrays presented similar performance (Figure S19).

To evaluate the specificity of candidate markers, we included an additional 44 patients without CRC, including benign or malignant breast (N = 20) or lung cancer (N = 24). There was no significant difference in plasma cfDNA concentration between non-CRC patients and healthy controls (Figure S18). Furthermore, a few copies of methylated molecules were detected in approximately 1/3 of the samples, which was significantly lower than the detection rate in cfDNA samples from CRC patients (Figure 4A). The AUCs of the three mddPCR assays for distinguishing non-CRC patients from healthy controls were 0.599 (95% CI: 0.527-0.671), 0.588 (95% CI: 0.521-0.656), and 0.541 (95% CI: 0.487-0.595), indicating that these markers are only specific for diagnosing CRC to a certain extent.

To avoid the confusion caused by benign diseases, the diagnostic efficacy of mddPCR assays in breast cancer and lung cancer were evaluated after benign diseases were excluded, and the AUC values of the three mddPCR assays were 0.587 (95% CI: 0.506-0.668), 0.618 (95% CI: 0.533-0.702) and 0.550 (95% CI: 0.485-0.616), respectively. The three mddPCR assays had poor diagnostic efficacy in detecting cancers other than CRC.

**Supplementary References**

1. Luo Y, Wong CJ, Kaz AM, et al. Differences in DNA methylation signatures reveal multiple pathways of progression from adenoma to colorectal cancer. *Gastroenterology*. Aug 2014;147(2):418-29.e8. doi:10.1053/j.gastro.2014.04.039

2. Barrow TM, Klett H, Toth R, et al. Smoking is associated with hypermethylation of the APC 1A promoter in colorectal cancer: the ColoCare Study. *The Journal of pathology*. Nov 2017;243(3):366-375. doi:10.1002/path.4955

3. Díez-Villanueva A, Sanz-Pamplona R, Carreras-Torres R, et al. DNA methylation events in transcription factors and gene expression changes in colon cancer. *Epigenomics*. Sep 2020;12(18):1593-1610. doi:10.2217/epi-2020-0029

4. Qu X, Sandmann T, Frierson H, Jr., et al. Integrated genomic analysis of colorectal cancer progression reveals activation of EGFR through demethylation of the EREG promoter. *Oncogene*. Dec 15 2016;35(50):6403-6415. doi:10.1038/onc.2016.170

5. Fan J, Li J, Guo S, et al. Genome-wide DNA methylation profiles of low- and high-grade adenoma reveals potential biomarkers for early detection of colorectal carcinoma. *Clin Epigenetics*. Apr 21 2020;12(1):56. doi:10.1186/s13148-020-00851-3

6. Legendre CR, Demeure MJ, Whitsett TG, et al. Pathway Implications of Aberrant Global Methylation in Adrenocortical Cancer. *PLoS One*. 2016;11(3):e0150629. doi:10.1371/journal.pone.0150629

7. Fujimoto M, Arai E, Tsumura K, et al. Establishment of diagnostic criteria for upper urinary tract urothelial carcinoma based on genome-wide DNA methylation analysis. *Epigenetics*. Dec 2020;15(12):1289-1301. doi:10.1080/15592294.2020.1767374

8. Concepcion D, Ross KD, Hutt KR, Yeo GW, Hamilton BA. Nxf1 natural variant E610G is a semi-dominant suppressor of IAP-induced RNA processing defects. *PLoS Genet*. Apr 2015;11(4):e1005123. doi:10.1371/journal.pgen.1005123

9. Teschendorff AE, Jones A, Widschwendter M. Stochastic epigenetic outliers can define field defects in cancer. *BMC Bioinformatics*. Apr 22 2016;17:178. doi:10.1186/s12859-016-1056-z

10. Lando M, Fjeldbo CS, Wilting SM, et al. Interplay between promoter methylation and chromosomal loss in gene silencing at 3p11-p14 in cervical cancer. *Epigenetics*. 2015;10(10):970-80. doi:10.1080/15592294.2015.1085140

11. Chan-On W, Nairismägi ML, Ong CK, et al. Exome sequencing identifies distinct mutational patterns in liver fluke-related and non-infection-related bile duct cancers. *Nat Genet*. Dec 2013;45(12):1474-8. doi:10.1038/ng.2806

12. Soares-Lima SC, Mehanna H, Camuzi D, et al. Upper Aerodigestive Tract Squamous Cell Carcinomas Show Distinct Overall DNA Methylation Profiles and Different Molecular Mechanisms behind WNT Signaling Disruption. *Cancers (Basel)*. Jun 16 2021;13(12)doi:10.3390/cancers13123014

13. Hao JJ, Lin DC, Dinh HQ, et al. Spatial intratumoral heterogeneity and temporal clonal evolution in esophageal squamous cell carcinoma. *Nat Genet*. Dec 2016;48(12):1500-1507. doi:10.1038/ng.3683

14. De Meyer T, Bady P, Trooskens G, et al. Genome-wide DNA methylation detection by MethylCap-seq and Infinium HumanMethylation450 BeadChips: an independent large-scale comparison. *Sci Rep*. Oct 20 2015;5:15375. doi:10.1038/srep15375

15. Sturm D, Witt H, Hovestadt V, et al. Hotspot mutations in H3F3A and IDH1 define distinct epigenetic and biological subgroups of glioblastoma. *Cancer Cell*. Oct 16 2012;22(4):425-37. doi:10.1016/j.ccr.2012.08.024

16. Lim AM, Wong NC, Pidsley R, et al. Genome-scale methylation assessment did not identify prognostic biomarkers in oral tongue carcinomas. *Clin Epigenetics*. 2016;8:74. doi:10.1186/s13148-016-0235-0

17. Wei JH, Haddad A, Wu KJ, et al. A CpG-methylation-based assay to predict survival in clear cell renal cell carcinoma. *Nature communications*. Oct 30 2015;6:8699. doi:10.1038/ncomms9699

18. Davegårdh C, Säll J, Benrick A, et al. VPS39-deficiency observed in type 2 diabetes impairs muscle stem cell differentiation via altered autophagy and epigenetics. *Nature communications*. Apr 23 2021;12(1):2431. doi:10.1038/s41467-021-22068-5

19. Sandoval J, Mendez-Gonzalez J, Nadal E, et al. A prognostic DNA methylation signature for stage I non-small-cell lung cancer. *J Clin Oncol*. Nov 10 2013;31(32):4140-7. doi:10.1200/jco.2012.48.5516

20. Endo Y, Fujimoto M, Ito N, et al. Clinicopathological impacts of DNA methylation alterations on pancreatic ductal adenocarcinoma: prediction of early recurrence based on genome-wide DNA methylation profiling. *J Cancer Res Clin Oncol*. May 2021;147(5):1341-1354. doi:10.1007/s00432-021-03541-6

21. Nones K, Waddell N, Song S, et al. Genome-wide DNA methylation patterns in pancreatic ductal adenocarcinoma reveal epigenetic deregulation of SLIT-ROBO, ITGA2 and MET signaling. *Int J Cancer*. Sep 1 2014;135(5):1110-8. doi:10.1002/ijc.28765

22. Letouzé E, Martinelli C, Loriot C, et al. SDH mutations establish a hypermethylator phenotype in paraganglioma. *Cancer Cell*. Jun 10 2013;23(6):739-52. doi:10.1016/j.ccr.2013.04.018

23. Aref-Eshghi E, Schenkel LC, Ainsworth P, et al. Genomic DNA Methylation-Derived Algorithm Enables Accurate Detection of Malignant Prostate Tissues. *Front Oncol*. 2018;8:100. doi:10.3389/fonc.2018.00100

24. Guilhamon P, Eskandarpour M, Halai D, et al. Meta-analysis of IDH-mutant cancers identifies EBF1 as an interaction partner for TET2. *Nature communications*. 2013;4:2166. doi:10.1038/ncomms3166

25. Conway K, Edmiston SN, Parker JS, et al. Identification of a Robust Methylation Classifier for Cutaneous Melanoma Diagnosis. *J Invest Dermatol*. Jun 2019;139(6):1349-1361. doi:10.1016/j.jid.2018.11.024

26. Ooi WF, Xing M, Xu C, et al. Epigenomic profiling of primary gastric adenocarcinoma reveals super-enhancer heterogeneity. *Nature communications*. Sep 28 2016;7:12983. doi:10.1038/ncomms12983

27. Braun SMG, Petrova R, Tang J, et al. BAF subunit switching regulates chromatin accessibility to control cell cycle exit in the developing mammalian cortex. *Genes Dev*. Mar 1 2021;35(5-6):335-353. doi:10.1101/gad.342345.120

28. Killian JK, Dorssers LC, Trabert B, et al. Imprints and DPPA3 are bypassed during pluripotency- and differentiation-coupled methylation reprogramming in testicular germ cell tumors. *Genome Res*. Nov 2016;26(11):1490-1504. doi:10.1101/gr.201293.115

29. Bisarro Dos Reis M, Barros-Filho MC, Marchi FA, et al. Prognostic Classifier Based on Genome-Wide DNA Methylation Profiling in Well-Differentiated Thyroid Tumors. *J Clin Endocrinol Metab*. Nov 1 2017;102(11):4089-4099. doi:10.1210/jc.2017-00881

30. Beltrami CM, Dos Reis MB, Barros-Filho MC, et al. Integrated data analysis reveals potential drivers and pathways disrupted by DNA methylation in papillary thyroid carcinomas. *Clin Epigenetics*. 2017;9:45. doi:10.1186/s13148-017-0346-2

31. Uhde K, van Tol HTA, Stout TAE, Roelen BAJ. MicroRNA Expression in Bovine Cumulus Cells in Relation to Oocyte Quality. *Noncoding RNA*. Mar 11 2017;3(1)doi:10.3390/ncrna3010012

32. Teschendorff AE, Yang Z, Wong A, et al. Correlation of Smoking-Associated DNA Methylation Changes in Buccal Cells With DNA Methylation Changes in Epithelial Cancer. *JAMA Oncol*. Jul 2015;1(4):476-85. doi:10.1001/jamaoncol.2015.1053

33. Gotoh O, Sugiyama Y, Takazawa Y, et al. Clinically relevant molecular subtypes and genomic alteration-independent differentiation in gynecologic carcinosarcoma. *Nature communications*. Oct 31 2019;10(1):4965. doi:10.1038/s41467-019-12985-x

34. Chen X, Gole J, Gore A, et al. Non-invasive early detection of cancer four years before conventional diagnosis using a blood test. *Nature communications*. Jul 21 2020;11(1):3475. doi:10.1038/s41467-020-17316-z

35. Hajian-Tilaki K. Sample size estimation in diagnostic test studies of biomedical informatics. *J Biomed Inform*. Apr 2014;48:193-204. doi:10.1016/j.jbi.2014.02.013

# Table S1. Public and inhouse datasets used in this study.

| **Dataset source** | **Sample source** | **Sample type** | **Platform** | **Number of samples** |
| --- | --- | --- | --- | --- |
| **Discovery cohort (N = 10,315)** | | | | |
| TCGA | CRC | Tissue | 450K array | Tumor = 395, Normal = 45 |
| TCGA | ACC | Tissue | 450K array | Tumor = 80, Normal = 0 |
| TCGA | BLCA | Tissue | 450K array | Tumor = 412, Normal = 21 |
| TCGA | BRCA | Tissue | 450K array | Tumor = 785, Normal = 98 |
| TCGA | CESC | Tissue | 450K array | Tumor = 307, Normal = 3 |
| TCGA | CHOL | Tissue | 450K array | Tumor = 36, Normal = 9 |
| TCGA | ESCA | Tissue | 450K array | Tumor = 185, Normal = 16 |
| TCGA | GBMLGG | Tissue | 450K array | Tumor = 656, Normal = 2 |
| TCGA | HNSC | Tissue | 450K array | Tumor = 528, Normal = 50 |
| TCGA | KICH | Tissue | 450K array | Tumor = 66, Normal = 0 |
| TCGA | KIRC | Tissue | 450K array | Tumor = 319, Normal = 160 |
| TCGA | KIRP | Tissue | 450K array | Tumor = 275, Normal = 45 |
| TCGA | LIHC | Tissue | 450K array | Tumor = 377, Normal = 50 |
| TCGA | LUAD | Tissue | 450K array | Tumor = 458, Normal = 32 |
| TCGA | LUSC | Tissue | 450K array | Tumor = 372, Normal = 43 |
| TCGA | MESO | Tissue | 450K array | Tumor = 87, Normal = 0 |
| TCGA | OV | Tissue | 450K array | Tumor = 10, Normal = 0 |
| TCGA | PAAD | Tissue | 450K array | Tumor = 184, Normal = 10 |
| TCGA | PCPG | Tissue | 450K array | Tumor = 179, Normal = 3 |
| TCGA | PRAD | Tissue | 450K array | Tumor = 498, Normal = 50 |
| TCGA | SARC | Tissue | 450K array | Tumor = 261, Normal = 4 |
| TCGA | SKCM | Tissue | 450K array | Tumor = 105, Normal = 2 |
| TCGA | STAD | Tissue | 450K array | Tumor = 396, Normal = 2 |
| TCGA | TGCT | Tissue | 450K array | Tumor = 150, Normal = 0 |
| TCGA | THCA | Tissue | 450K array | Tumor = 507, Normal = 56 |
| TCGA | THYM | Tissue | 450K array | Tumor = 124, Normal = 2 |
| TCGA | UCEC | Tissue | 450K array | Tumor = 431, Normal = 46 |
| TCGA | UCS | Tissue | 450K array | Tumor = 57, Normal = 0 |
| TCGA | UVM | Tissue | 450K array | Tumor = 80, Normal = 0 |
| GSE51032* | Healthy | White blood cells | 450K array | Normal = 590 |
| GSE40279 | Healthy | White blood cells | 450K array | Normal = 656 |
| **Validation cohort (N = 5,604)** | | | | |
| GSE48684^†^ & GSE101764 & GSE131013 | CRC | Tissue | 450K array | Tumor = 270, Normal = 334 |
| GSE48684^†^ & GSE77954 & GSE139404 | Adenoma | Tissue | 450K array | Adenoma = 94, Normal = 72 |
| GSE77871 | ACC | Tissue | 450K array | Tumor = 18, Normal = 6 |
| GSE111933 | BLCA | Tissue | 450K array | Tumor = 46, Normal = 46 |
| GSE66695 & GSE69914 | BRCA | Tissue | 450K array | Tumor = 395, Normal = 132 |
| GSE68339 | CESC | Tissue | 450K array | Tumor = 270 |
| GSE49656 | CHOL | Tissue | 450K array | Tumor = 32, Normal = 4 |
| GSE178212 & GSE79366 | ESCA | Tissue | 450K array | Tumor = 36, Normal = 18 |
| GSE60274 & GSE36278 | GBMLGG | Tissue | 450K array | Tumor = 208, Normal = 11 |
| GSE79556 | HNSC | Tissue | 450K array | Tumor = 83 |
| GSE61441 | KIRC | Tissue | 450K array | Tumor = 46, Normal = 46 |
| GSE157341 | LIHC | Tissue | 450K array | Tumor = 239, Normal = 35 |
| GSE39279 | LUAD | Tissue | 450K array | Tumor = 322 |
| GSE39279 | LUSC | Tissue | 450K array | Tumor = 122 |
| GSE155353 & GSE49149 | PAAD | Tissue | 450K array | Tumor = 249, Normal = 62 |
| GSE43293 | PCPG | Tissue | 450K array | Tumor = 22, Normal = 2 |
| GSE112047 | PRAD | Tissue | 450K array | Tumor = 31, Normal = 16 |
| GSE40853 | SARC | Tissue | 450K array | Tumor = 51 |
| GSE120878 | SKCM | Tissue | 450K array | Tumor = 89, Normal = 73 |
| GSE85464 & GSE164988 | STAD | Tissue | 450K array | Tumor = 31, Normal = 31 |
| GSE74104 | TGCT | Tissue | 450K array | Tumor = 130, Normal = 128 |
| GSE97466 & GSE86961 | THCA | Tissue | 450K array | Tumor = 115, Normal = 108 |
| GSE94769 | THYM | Tissue | 450K array | Tumor = 21, Normal = 1 |
| GSE67116 | UCEC | Tissue | 450K array | Tumor = 86, Normal = 8 |
| GSE136790 | UCS | Tissue | 450K array | Tumor = 88 |
| CCLE | 33 cancer types | Cell lines | RRBS/RNA-seq | Tumor = 1,377 |
| **Inhouse validation cohort (N = 372)** |  |  |  |  |
| In-house study | CRC | Tissue | Targeted bisulfite sequencing | Tumor = 276, Normal = 24 |
| In-house study | CRC | White blood cells | Targeted bisulfite sequencing | Tumor = 29, Normal = 29 |
| In-house study | CRC | cfDNA | Targeted bisulfite sequencing | Tumor = 9, Normal = 5 |
| **cfDNA Validation cohort (N = 370)** | | | | |
| In-house study | CRC | cfDNA | Droplet digital PCR | Normal = 103, Tumor = 195 |
| In-house study | Adenoma/Polyp | cfDNA | Droplet digital PCR | Polyp = 6, Adenoma = 22 |
| In-house study | Breast lesion | cfDNA | Droplet digital PCR | Benign = 6, Tumor = 14 |
| In-house study | Lung lesion | cfDNA | Droplet digital PCR | Benign = 6, Tumor = 18 |

Note: * GSE51032 contains data from 845 participants in the EPIC-Italy cohort. At the last follow-up (2010), 424 participants remained cancer-free and 166 had developed primary colorectal cancer. ^†^ The normal samples in GSE48684 were shared in both two group. cfDNA, Cell-free DNA;

# Table S2. Primer sequences of candidate CRC-specific methylation markers for targeted bisulfite sequencing.

| **CpG sites** | **Gene** | **Sample type** | **Primer** | **Primer sequence (5'-3')** | **Strand** | **Genomic position ^†^** | **Amplicon length (bp)** |
| --- | --- | --- | --- | --- | --- | --- | --- |
| cg20841906 | *B3GALNT1* | Tissue & WBC | Forward | TTTTAGAGGAGTGGGYGGTGT | + | chr3:160822863-160823063 | 201 |
|  |  |  | Reverse | ACCTTCCCRCTTCCRAACTAAA |  |  |  |
| cg00117463 | *C6orf97* | Tissue & WBC | Forward | ATATGGTTGGTGTAGTTTAGGTTTATG | - | chr6:151815289-151815086 | 204 |
|  |  |  | Reverse | AATCCACCCCRACCTCCT |  |  |  |
| cg18174928 | *LIFR* | Tissue & WBC | Forward | GAYGGTTTTGYGGGGAGGA | + | chr5: 38557063-38557297 | 235 |
|  |  |  | Reverse | CCCCACACCCRACAAAAA |  |  |  |
| cg26970847 | *ZNF264* | Tissue & WBC | Forward | TTTGTTYGGTTTTGATTAGGGATTT | - | chr19: 57702959-57702709 | 251 |
|  |  |  | Reverse | TTCCTCCCRTTATTAAACACATAAAA |  |  |  |
| cg11140785 | *ZNF264* | Tissue & WBC | Forward | TTTGYGGTTTAGGGGGTGA | + | chr19: 57703259- 57703494 | 236 |
|  |  |  | Reverse | TTCCAATAAACCCAACACTCCT |  |  |  |
| cg14786398 | *ZNF543* | Tissue & WBC | Forward | AGGGAAAAATTGTTTTATTTAGAAGGT | + | chr19: 57831716- 57831880 | 165 |
|  |  |  | Reverse | TTCCAAAACCAATCCTACTCAC |  |  |  |
|  |  | cfDNA | Forward | GTTAATGAYGGGTTAGGATA | + | chr19: 57831773- 57831873 | 101 |
|  |  |  | Reverse | ACCAATCCTACTCACRAAAATAA |  |  |  |

Note: ^†^ The genomic positions based on the GRCh37/hg19 coordinates, and the degenerate bases Y: C/T and R: A/G. WBC, White blood cell; cfDNA, Cell-free DNA;

# Table S3. Primer and probe sequences of candidate CRC-specific methylation markers and reference gene in ddPCR.

| **Gene/Region** | **CpG sites** | **Primer** | **Primer sequence (5'-3')** | **Strand** | **Genomic location** | **Product size (bp)** |
| --- | --- | --- | --- | --- | --- | --- |
| FAM72A | cg00552973 | Universal forward | GGGATAATTGGAGTTGTATAYGTTYG | + | chr1: 206,137,016-206,137,085 | 70 |
|  |  | Universal reverse | CCCTCACCTCTAAAACAACCTTC |  |  |  |
|  |  | Methylated probe | FAM-TACCGCCTCCCGC-MGB |  |  |  |
| FAM72B_1 | cg07784526 | Universal forward | TYGGTGTTTTAGTTTTTGGAGGA | + | chr1: 120,838,292-120,838,354 | 63 |
|  | cg18948743 | Methylated reverse | CCCGACGAAATCCTACGAA |  |  |  |
|  |  | Methylated probe | FAM-ACGACGAAAACTC-MGB |  |  |  |
| FAM72B_2 | cg00198436 | Universal forward | GATGTTTAGTTYGAGATTYGATAGTAGGG |  | chr1:120,839,348-120,839,424 | 77 |
|  | cg07236150 | Universal reverse | AAACATCCCTTCCCCAACATTA |  |  |  |
|  | cg09169215 | Methylated probe | FAM-CCGAAACGCTCCG-MGB |  |  |  |
| LIFR | cg18174928 | Universal forward | YGAGAAAGGTYGAGTGYGYGAYG | + | chr5: 38557044-38557130 | 87 |
|  |  | Universal reverse | CAAAACACTATTTACAACRACAACAAC |  |  |  |
|  |  | Methylated probe | FAM–ACGCGATCCTCCCCGCAA-BHQ1 |  |  |  |
| OSMR | cg17528648 | Methylated forward | TCGCGTTTGTTTCGTAGTTGA | + | chr5: 38,846,099-38,846,167 | 69 |
|  |  | Universal reverse | CRAACTCCRAAACRAACRTACAA |  |  |  |
|  |  | Methylated probe | FAM–AACGACCCGAACCG-MGB |  |  |  |
| ZNF264 | cg11140785 | Universal forward | GYGGTTTAGGGGGTGAYGTG | + | chr19: 57,703,262-57,703,328 | 67 |
|  |  | Universal reverse | CACCRTCCACTCACCTAAACCC |  |  |  |
|  |  | Methylated probe | FAM–CCGTCAACACCGCT–MGB |  |  |  |
| ZNF543 | cg14786398 | Universal forward | GGATAYGGGYGATTTTTGYGG | + | chr19: 57,831,788-57,831,863 | 76 |
|  |  | Universal reverse | CTCACRAAAATAACRTCACRCRA |  |  |  |
|  |  | Methylated probe | FAM-TCCGCCGCCCTAA-MGB |  |  |  |
| ACTB |  | Forward | GGTTAAGTGTGATTTTGTGGTGTG | - | chr7: 5,566,778-5,566,699 | 80 |
|  |  | Reverse | CCTTTTACAAAATTCACCCTCCT |  |  |  |
|  |  | Probe | VIC-ACCCTCTACTACCC-MGB |  |  |  |

Note: The genomic positions refer to GRCh37/hg19 coordinates, and the degenerate bases Y: C/T and R: A/G. ddPCR, Droplet digital PCR

# Table S4. Composition of the three multiplex droplet digital PCR assays.

|  | **Multiplex ddPCR assay 1** | | |  | **Multiplex ddPCR assay 2** | |  | **Multiplex ddPCR assay 3** | |
| --- | --- | --- | --- | --- | --- | --- | --- | --- | --- |
| Target region | LIFR | ZNF264 | ZNF543 |  | FAM72A | FAM72B_2 |  | FAM72B_1 | OSMR |
| Forward (20 μM) | 0.2 | 0.2 | 0.6 |  | 0.6 | 0.6 |  | 0.3 | 0.6 |
| Reverse (20 μM) | 0.2 | 0.2 | 0.6 |  | 0.6 | 0.6 |  | 0.3 | 0.6 |
| Taqman probe (10 μM) | 0.3 | 0.3 | 0.4 |  | 0.3 | 0.4 |  | 0.3 | 0.3 |
| ACTB (reference gene) |  | | |  |  | |  |  | |
| Forward (20 μM) | 0.2 | | |  | 0.3 | |  | 0.3 | |
| Reverse (20 μM) | 0.2 | | |  | 0.3 | |  | 0.3 | |
| Taqman probe (10 μM) | 0.2 | | |  | 0.2 | |  | 0.2 | |
| 2× ddPCR Supermix for Probes | 10 | | |  | 10 | |  | 10 | |
| Template DNA | 7.4 | | |  | 7.1 | |  | 7.8 | |
| Total volume | 21 | | |  | 21 | |  | 21 | |

# Table S5. The genomic characteristics of 15 CRC-specific methylation CpG sites.

| **CpG sites** | **Gene symbol** | **Gene description** | **Genomic coordinate** | **Strand** | **Relation to Island** | **UCSC refGene group** | **TSS** | **mRNA** | **mRNA**  **strand** | **Distance from TSS** | **Evolutionary conservation score** |
| --- | --- | --- | --- | --- | --- | --- | --- | --- | --- | --- | --- |
| cg20841906 | *B3GALNT1* | Beta-1,3-N-Acetylgalactosaminyltransferase 1 | chr3: 160822911 | - | Island | 5'UTR | 160823160 | NM_001038628 | - | 249 | 0.131596 |
| cg00117463 | *C6orf97* | chromosome 6 open reading frame 97 | chr6: 151815241 | + | Island | 5'UTR | 151815174 | NM_025059 | + | 67 | 0.0421392 |
| cg00552973 | *FAM72A* | Family With Sequence Similarity 72 Member A | chr1: 206137054 | - | Island | NA | 206138910 | NM_001123168 | + | -1856 | 0.358124 |
| cg10439914 | *FAM72A* |  | chr1: 206137759 | + | Island | TSS1500 |  |  |  | 1151 |  |
| cg07784526 | *FAM72B* | Family With Sequence Similarity 72 Member B | chr1: 120838320 | + | Island | TSS1500 | 120839004 | NM_001100910 | + | -684 | 0.339953 |
| cg18948743 | *FAM72B* |  | chr1: 120838323 | + | Island | TSS1500 |  |  |  | -681 |  |
| cg08728856 | *FAM72B* |  | chr1: 120838718 | + | Island | TSS1500 |  |  |  | -286 |  |
| cg00198436 | *FAM72B* |  | chr1: 120839380 | - | Island | 1stExon |  |  |  | 376 |  |
| cg07236150 | *FAM72B* |  | chr1: 120839385 | - | Island | 1stExon |  |  |  | 381 |  |
| cg09169215 | *FAM72B* |  | chr1: 120839390 | - | Island | 1stExon |  |  |  | 386 |  |
| cg18174928 | *LIFR* | LIF receptor alpha | chr5: 38557085 | + | Island | TSS1500 | 38556748 | NM_001127671 | - | -337 | 0.0599912 |
| cg17528648 | *OSMR* | Oncostatin M Receptor | chr5: 38846100 | + | Island | 5'UTR | 38846100 | NM_003999 | + | 0 | -0.0121864 |
| cg26970847 | *ZNF264* | Zinc Finger Protein 264 | chr19: 57702793 | - | Island | 1stExon | 57702867 | NM_003417 | + | -74 | -0.0938474 |
| cg11140785 | *ZNF264* |  | chr19: 57703301 | - | Island | TSS200 |  |  |  | 434 |  |
| cg14786398 | *ZNF543* | Zinc Finger Protein 543 | chr19: 57831816 | - | Island | TSS200 | 57831864 | NM_213598 | + | -48 | -0.206065 |

Note: The genomic positions refer to GRCh37/hg19 coordinates. TSS: Transcription Start Site.

# Table S6. The genomic characteristics of 12 CpG sites of *SEPT9* island.

| **CpG sites** | **Gene symbol** | **Gene description** | **Genomic coordinate** | **Strand** | **Relation to Island** | **UCSC refGene group** | **TSS** | **mRNA** | **mRNA**  **strand** | **Distance from TSS** | **Evolutionary conservation score** |
| --- | --- | --- | --- | --- | --- | --- | --- | --- | --- | --- | --- |
| cg22112360 | *SEPT9* | Septin 9 | chr17: 75368750 | + | Island | TSS1500 | 75277491 | NM_001113491 | + | 91259 | -0.222401 |
| cg06848185 | *SEPT9* |  | chr17: 75368902 | - | Island | TSS1500 |  |  |  | 91411 |  |
| cg19554255 | *SEPT9* |  | chr17: 75369051 | - | Island | TSS1500 |  |  |  | 91560 |  |
| cg16779463 | *SEPT9* |  | chr17: 75369055 | - | Island | TSS1500 |  |  |  | 91564 |  |
| cg17300544 | *SEPT9* |  | chr17: 75369091 | + | Island | TSS200 |  |  |  | 91600 |  |
| cg03804136 | *SEPT9* |  | chr17: 75369219 | + | Island | TSS200 |  |  |  | 91728 |  |
| cg15044248 | *SEPT9* |  | chr17: 75369224 | + | Island | TSS200 |  |  |  | 91733 |  |
| cg02884239 | *SEPT9* |  | chr17: 75369228 | + | Island | TSS200 |  |  |  | 91737 |  |
| cg20275528 | *SEPT9* |  | chr17: 75369484 | - | Island | 5'UTR |  |  |  | 91993 |  |
| cg12783819 | *SEPT9* |  | chr17: 75369657 | - | Island | 5'UTR |  |  |  | 92166 |  |
| cg05184938 | *SEPT9* |  | chr17: 75369939 | - | Island | 5'UTR |  |  |  | 92448 |  |
| cg02320862 | *SEPT9* |  | chr17: 75370284 | - | Island | 5'UTR |  |  |  | 92793 |  |

Note: The genomic positions refer to GRCh37/hg19 coordinates. TSS: Transcription Start Site.

# Table S7. Diagnostic performance analyses of 15 CpG sites for distinguishing colorectal cancer from adjacent normal tissues in four datasets.

| **CpG sites** | **Cut-off** | **AUC** | **TCGA** | | | | **GSE48684** | | | | **GSE101764** | | | | **GSE131013** | | | |
| --- | --- | --- | --- | --- | --- | --- | --- | --- | --- | --- | --- | --- | --- | --- | --- | --- | --- | --- |
|  |  |  | **TP** | **Sen** | **TN** | **Spe** | **TP** | **Sen** | **TN** | **Spe** | **TP** | **Sen** | **TN** | **Spe** | **TP** | **Sen** | **TN** | **Spe** |
| cg20841906 | 0.078 | 0.687 | 198 | 0.501 | 45 | 1.000 | 30 | 0.469 | 40 | 0.976 | 62 | 0.564 | 148 | 0.993 | 53 | 0.552 | 137 | 0.951 |
| cg00117463 | 0.202 | 0.643 | 176 | 0.446 | 45 | 1.000 | 24 | 0.375 | 41 | 1.000 | 50 | 0.455 | 149 | 1.000 | 39 | 0.406 | 140 | 0.972 |
| cg00552973 | 0.185 | 0.865 | 312 | 0.790 | 45 | 1.000 | 40 | 0.619 | 41 | 1.000 | 93 | 0.845 | 147 | 0.987 | 80 | 0.833 | 132 | 0.917 |
| cg10439914 | 0.110 | 0.855 | 287 | 0.727 | 45 | 1.000 | 36 | 0.562 | 40 | 0.976 | 91 | 0.827 | 147 | 0.987 | 78 | 0.812 | 131 | 0.910 |
| cg07784526 | 0.372 | 0.702 | 233 | 0.590 | 45 | 1.000 | 29 | 0.453 | 41 | 1.000 | 67 | 0.609 | 147 | 0.987 | 61 | 0.635 | 139 | 0.965 |
| cg18948743 | 0.286 | 0.723 | 241 | 0.610 | 45 | 1.000 | 33 | 0.516 | 39 | 0.951 | 69 | 0.627 | 143 | 0.960 | 61 | 0.632 | 137 | 0.951 |
| cg08728856 | 0.138 | 0.859 | 320 | 0.810 | 44 | 0.978 | 48 | 0.750 | 34 | 0.829 | 87 | 0.791 | 147 | 0.987 | 79 | 0.823 | 134 | 0.931 |
| cg00198436 | 0.158 | 0.863 | 310 | 0.785 | 44 | 0.978 | 45 | 0.703 | 36 | 0.878 | 80 | 0.727 | 148 | 0.993 | 75 | 0.781 | 136 | 0.944 |
| cg07236150 | 0.105 | 0.862 | 299 | 0.757 | 45 | 1.000 | 38 | 0.594 | 41 | 1.000 | 87 | 0.791 | 147 | 0.987 | 75 | 0.781 | 136 | 0.944 |
| cg09169215 | 0.134 | 0.829 | 292 | 0.739 | 45 | 1.000 | 38 | 0.594 | 41 | 1.000 | 86 | 0.782 | 141 | 0.946 | 73 | 0.760 | 136 | 0.944 |
| cg18174928 | 0.209 | 0.807 | 300 | 0.759 | 45 | 1.000 | 39 | 0.609 | 41 | 1.000 | 73 | 0.664 | 149 | 1.000 | 75 | 0.781 | 139 | 0.965 |
| cg17528648 | 0.296 | 0.903 | 323 | 0.818 | 45 | 1.000 | 34 | 0.531 | 41 | 1.000 | 91 | 0.827 | 131 | 0.879 | 85 | 0.885 | 128 | 0.889 |
| cg26970847 | 0.069 | 0.702 | 165 | 0.418 | 44 | 0.978 | 21 | 0.328 | 41 | 1.000 | 62 | 0.564 | 144 | 0.966 | 55 | 0.573 | 140 | 0.972 |
| cg11140785 | 0.066 | 0.765 | 208 | 0.527 | 45 | 1.000 | 23 | 0.359 | 41 | 1.000 | 65 | 0.591 | 147 | 0.987 | 60 | 0.625 | 140 | 0.972 |
| cg14786398 | 0.176 | 0.713 | 231 | 0.585 | 45 | 1.000 | 26 | 0.406 | 41 | 1.000 | 49 | 0.445 | 149 | 1.000 | 68 | 0.708 | 140 | 0.972 |

Note: TP, Number of true positive; Sen, Sensitivity; TN, Number of true negative; Spe, Specificity.

# Table S8. Diagnostic performance analyses of 11 CpG sites of *SEPT9* island for distinguishing colorectal cancer from adjacent normal tissues in four datasets.

| **CpG sites** | **Cut-off** | **AUC** | **TCGA** | | | | **GSE48684** | | | | **GSE101764** | | | | **GSE131013** | | | |
| --- | --- | --- | --- | --- | --- | --- | --- | --- | --- | --- | --- | --- | --- | --- | --- | --- | --- | --- |
|  |  |  | **TP** | **Sen** | **TN** | **Spe** | **TP** | **Sen** | **TN** | **Spe** | **TP** | **Sen** | **TN** | **Spe** | **TP** | **Sen** | **TN** | **Spe** |
| cg06848185 | 0.127 | 0.890 | 307 | 0.777 | 44 | 0.978 | 40 | 0.625 | 40 | 0.975 | 80 | 0.727 | 147 | 0.987 | 75 | 0.781 | 140 | 0.972 |
| cg19554255 | 0.119 | 0.919 | 323 | 0.818 | 44 | 0.978 | 52 | 0.812 | 39 | 0.951 | 79 | 0.718 | 147 | 0.987 | 77 | 0.802 | 140 | 0.972 |
| cg16779463 | 0.084 | 0.846 | 288 | 0.729 | 44 | 0.978 | 35 | 0.547 | 40 | 0.976 | 80 | 0.727 | 145 | 0.973 | 75 | 0.781 | 140 | 0.972 |
| cg17300544 | 0.105 | 0.891 | 297 | 0.752 | 44 | 0.978 | 40 | 0.625 | 38 | 0.927 | 81 | 0.736 | 144 | 0.966 | 75 | 0.779 | 139 | 0.965 |
| cg03804136 | 0.133 | 0.848 | 287 | 0.727 | 45 | 1.000 | 38 | 0.594 | 40 | 0.976 | 79 | 0.718 | 144 | 0.966 | 75 | 0.781 | 140 | 0.972 |
| cg15044248 | 0.066 | 0.872 | 307 | 0.777 | 44 | 0.978 | 41 | 0.641 | 40 | 0.976 | 83 | 0.755 | 140 | 0.940 | 76 | 0.792 | 139 | 0.965 |
| cg02884239 | 0.140 | 0.857 | 288 | 0.729 | 44 | 0.978 | 46 | 0.719 | 39 | 0.951 | 75 | 0.682 | 147 | 0.987 | 73 | 0.760 | 138 | 0.958 |
| cg20275528 | 0.413 | 0.967 | 373 | 0.944 | 45 | 1.000 | 45 | 0.703 | 41 | 1.000 | 95 | 0.864 | 145 | 0.973 | 84 | 0.875 | 136 | 0.944 |
| cg12783819 | 0.477 | 0.907 | 351 | 0.889 | 40 | 0.889 | 43 | 0.672 | 36 | 0.878 | 87 | 0.791 | 124 | 0.832 | 79 | 0.823 | 114 | 0.792 |
| cg05184938 | 0.358 | 0.883 | 347 | 0.878 | 40 | 0.889 | 52 | 0.812 | 17 | 0.415 | 54 | 0.491 | 140 | 0.940 | 66 | 0.688 | 136 | 0.944 |
| cg02320862 | 0.624 | 0.876 | 328 | 0.830 | 42 | 0.933 | 45 | 0.703 | 27 | 0.659 | 32 | 0.291 | 145 | 0.973 | 74 | 0.766 | 125 | 0.866 |

Note: TP, Number of true positive; Sen, Sensitivity; TN, Number of true negative; Spe, Specificity. The cg22112360 with a missing value above 10% in the TCGA CRC dataset and removed.

# Table S9. Diagnostic performance analyses of 15 CpG sites for distinguishing adenoma from adjacent normal tissues in three datasets.

| **CpG sites** | **Cut-off** | **AUC** | **GSE48684** | | | | **GSE77954** | | | | **GSE139404** | | | |
| --- | --- | --- | --- | --- | --- | --- | --- | --- | --- | --- | --- | --- | --- | --- |
|  |  |  | **TP** | **Sen** | **TN** | **Spe** | **TP** | **Sen** | **TN** | **Spe** | **TP** | **Sen** | **TN** | **Spe** |
| cg20841906 | 0.078 | 0.647 | 13 | 0.310 | 40 | 0.976 | 8 | 0.667 | 11 | 1.000 | 16 | 0.400 | 17 | 0.850 |
| cg00117463 | 0.202 | 0.740 | 9 | 0.214 | 41 | 1.000 | 8 | 0.667 | 10 | 0.909 | 10 | 0.250 | 19 | 0.950 |
| cg00552973 | 0.185 | 0.861 | 27 | 0.643 | 41 | 1.000 | 12 | 1.000 | 10 | 0.909 | 27 | 0.675 | 17 | 0.850 |
| cg10439914 | 0.110 | 0.861 | 26 | 0.619 | 40 | 0.976 | 12 | 1.000 | 9 | 0.818 | 27 | 0.675 | 18 | 0.900 |
| cg07784526 | 0.372 | 0.782 | 18 | 0.429 | 41 | 1.000 | 11 | 0.917 | 10 | 0.909 | 22 | 0.550 | 18 | 0.900 |
| cg18948743 | 0.286 | 0.772 | 20 | 0.476 | 39 | 0.951 | 11 | 0.917 | 10 | 0.909 | 27 | 0.675 | 18 | 0.900 |
| cg08728856 | 0.138 | 0.836 | 34 | 0.810 | 34 | 0.829 | 12 | 1.000 | 6 | 0.545 | 28 | 0.700 | 18 | 0.900 |
| cg00198436 | 0.158 | 0.812 | 34 | 0.810 | 36 | 0.878 | 12 | 1.000 | 8 | 0.727 | 25 | 0.625 | 18 | 0.900 |
| cg07236150 | 0.105 | 0.843 | 27 | 0.634 | 41 | 1.000 | 12 | 1.000 | 8 | 0.727 | 25 | 0.625 | 18 | 0.900 |
| cg09169215 | 0.134 | 0.853 | 26 | 0.619 | 41 | 1.000 | 11 | 0.917 | 8 | 0.727 | 25 | 0.625 | 18 | 0.900 |
| cg18174928 | 0.209 | 0.855 | 29 | 0.690 | 41 | 1.000 | 10 | 0.833 | 10 | 0.909 | 20 | 0.500 | 16 | 0.800 |
| cg17528648 | 0.296 | 0.879 | 31 | 0.738 | 41 | 1.000 | 12 | 1.000 | 9 | 0.818 | 32 | 0.800 | 14 | 0.700 |
| cg26970847 | 0.069 | 0.788 | 20 | 0.476 | 41 | 1.000 | 10 | 0.833 | 5 | 0.455 | 21 | 0.525 | 19 | 0.950 |
| cg11140785 | 0.066 | 0.805 | 26 | 0.619 | 41 | 1.000 | 10 | 0.833 | 6 | 0.545 | 21 | 0.525 | 17 | 0.850 |
| cg14786398 | 0.176 | 0.719 | 19 | 0.452 | 41 | 1.000 | 9 | 0.75 | 10 | 0.909 | 19 | 0.475 | 16 | 0.800 |

Note: TP, Number of true positive; Sen, Sensitivity; TN, Number of true negative; Spe, Specificity.

# Table S10. Receiver operating characteristic curve analyses of 16 CpG sites in target region of *B3GALNT1* between colorectal cancer and adjacent normal tissues in our inhouse primary validation dataset.

| **Gene** | **Genomic Coordinate** | **AUC** | **Cut-off Value** | **Sensitivity (%)** | **Specificity (%)** |
| --- | --- | --- | --- | --- | --- |
| *B3GALNT1* | 160822888 | 0.755 | 0.066 | 53.7 | 100 |
| *B3GALNT1* | 160822900 | 0.806 | 0.027 | 61.2 | 95.8 |
| *B3GALNT1* | 160822911 * | 0.803 | 0.024 | 61.2 | 95.8 |
| *B3GALNT1* | 160822917 | 0.810 | 0.045 | 59.0 | 95.8 |
| *B3GALNT1* | 160822923 | 0.785 | 0.026 | 63.9 | 95.8 |
| *B3GALNT1* | 160822926 | 0.785 | 0.018 | 67.8 | 87.5 |
| *B3GALNT1* | 160822928 | 0.806 | 0.07 | 54.6 | 100 |
| *B3GALNT1* | 160822933 | 0.776 | 0.028 | 60.8 | 95.8 |
| *B3GALNT1* | 160822942 | 0.773 | 0.041 | 57.7 | 100 |
| *B3GALNT1* | 160822948 | 0.825 | 0.019 | 69.6 | 95.8 |
| *B3GALNT1* | 160822985 | 0.797 | 0.049 | 57.7 | 100 |
| *B3GALNT1* | 160822990 | 0.779 | 0.055 | 54.6 | 100 |
| *B3GALNT1* | 160823007 | 0.763 | 0.055 | 54.6 | 100 |
| *B3GALNT1* | 160823033 | 0.777 | 0.036 | 59.0 | 95.8 |
| *B3GALNT1* | 160823036 | 0.790 | 0.103 | 52.4 | 100 |
| *B3GALNT1* | 160823038 | 0.760 | 0.045 | 56.4 | 95.8 |

Note: * The target CpG of *B3GALNT1* (cg20841906) in the annotation information of Infinium 450K array.

# Table S11. Receiver operating characteristic curve analyses of 31 CpG sites in target region of *C6orf97* between colorectal cancer and adjacent normal tissues in our inhouse primary validation dataset.

| **Gene** | **Genomic Coordinate** | **AUC** | **Cut-off Value** | **Sensitivity (%)** | **Specificity (%)** |
| --- | --- | --- | --- | --- | --- |
| *C6orf97* | 151815261 | 0.625 | 0.035 | 46.3 | 95.8 |
| *C6orf97* | 151815256 | 0.633 | 0.029 | 42.7 | 95.8 |
| *C6orf97* | 151815250 | 0.65 | 0.034 | 47.1 | 95.8 |
| *C6orf97* | 151815244 | 0.683 | 0.042 | 44.1 | 95.8 |
| *C6orf97* | 151815242 * | 0.646 | 0.047 | 44.5 | 95.8 |
| *C6orf97* | 151815238 | 0.642 | 0.039 | 46.3 | 95.8 |
| *C6orf97* | 151815232 | 0.667 | 0.039 | 45.8 | 95.8 |
| *C6orf97* | 151815226 | 0.65 | 0.042 | 44.5 | 95.8 |
| *C6orf97* | 151815212 | 0.69 | 0.04 | 50.2 | 95.8 |
| *C6orf97* | 151815209 | 0.649 | 0.05 | 47.1 | 95.8 |
| *C6orf97* | 151815206 | 0.633 | 0.041 | 46.3 | 95.8 |
| *C6orf97* | 151815203 | 0.673 | 0.04 | 47.6 | 95.8 |
| *C6orf97* | 151815196 | 0.678 | 0.041 | 48.5 | 95.8 |
| *C6orf97* | 151815193 | 0.638 | 0.039 | 47.6 | 95.8 |
| *C6orf97* | 151815186 | 0.668 | 0.021 | 54.6 | 91.7 |
| *C6orf97* | 151815184 | 0.645 | 0.042 | 47.1 | 95.8 |
| *C6orf97* | 151815172 | 0.642 | 0.042 | 48.9 | 95.8 |
| *C6orf97* | 151815165 | 0.65 | 0.041 | 48.9 | 95.8 |
| *C6orf97* | 151815154 | 0.664 | 0.062 | 48.5 | 95.8 |
| *C6orf97* | 151815152 | 0.652 | 0.049 | 50.2 | 95.8 |
| *C6orf97* | 151815146 | 0.686 | 0.05 | 49.8 | 95.8 |
| *C6orf97* | 151815144 | 0.649 | 0.05 | 50.7 | 95.8 |
| *C6orf97* | 151815141 | 0.655 | 0.077 | 52.4 | 95.8 |
| *C6orf97* | 151815138 | 0.659 | 0.058 | 49.3 | 95.8 |
| *C6orf97* | 151815136 | 0.657 | 0.047 | 50.7 | 95.8 |
| *C6orf97* | 151815134 | 0.64 | 0.051 | 51.5 | 95.8 |
| *C6orf97* | 151815128 | 0.676 | 0.051 | 50.7 | 95.8 |
| *C6orf97* | 151815126 | 0.644 | 0.059 | 48.9 | 95.8 |
| *C6orf97* | 151815124 | 0.66 | 0.049 | 51.1 | 95.8 |
| *C6orf97* | 151815110 | 0.669 | 0.051 | 48.9 | 95.8 |
| *C6orf97* | 151815106 | 0.641 | 0.052 | 50.7 | 95.8 |

Note: * The target CpG of *C6orf97* (cg00117463) in the annotation information of Infinium 450K array.

# Table S12. Receiver operating characteristic curve analyses of 17 CpG sites in target region of *LIFR* between colorectal cancer and adjacent normal tissues in our inhouse primary validation dataset.

| **Gene** | **Genomic Coordinate** | **AUC** | **Cut-off Value** | **Sensitivity (%)** | **Specificity (%)** |
| --- | --- | --- | --- | --- | --- |
| *LIFR* | 38557083 | 0.824 | 0.109 | 66.5 | 95.8 |
| *LIFR* | 38557085 * | 0.823 | 0.161 | 61.2 | 100 |
| *LIFR* | 38557093 | 0.818 | 0.163 | 63.9 | 100 |
| *LIFR* | 38557099 | 0.790 | 0.121 | 65 | 95.8 |
| *LIFR* | 38557103 | 0.814 | 0.106 | 65.6 | 95.8 |
| *LIFR* | 38557112 | 0.805 | 0.108 | 65.5 | 95.8 |
| *LIFR* | 38557143 | 0.827 | 0.074 | 68.3 | 91.7 |
| *LIFR* | 38557151 | 0.790 | 0.107 | 57.3 | 100 |
| *LIFR* | 38557159 | 0.834 | 0.108 | 65.2 | 95.8 |
| *LIFR* | 38557162 | 0.841 | 0.162 | 61.7 | 100 |
| *LIFR* | 38557229 | 0.855 | 0.138 | 68.3 | 100 |
| *LIFR* | 38557253 | 0.84 | 0.141 | 65.2 | 100 |
| *LIFR* | 38557258 | 0.855 | 0.108 | 70.9 | 100 |
| *LIFR* | 38557261 | 0.878 | 0.118 | 70.5 | 100 |
| *LIFR* | 38557264 | 0.883 | 0.131 | 74 | 100 |
| *LIFR* | 38557268 | 0.853 | 0.113 | 71.8 | 100 |
| *LIFR* | 38557270 | 0.836 | 0.106 | 70.5 | 100 |

Note: * The target CpG of *LIFR* (cg18174928) in the annotation information of Infinium 450K array.

# Table S13. Receiver operating characteristic curve analyses of 36 CpG sites in target region of *ZNF264* between colorectal cancer and adjacent normal tissues in our inhouse primary validation dataset.

| **Gene** | **Genomic Coordinate** | **AUC** | **Cut-off Value** | **Sensitivity (%)** | **Specificity (%)** |
| --- | --- | --- | --- | --- | --- |
| *ZNF264* | 57702928 | 0.712 | 0.067 | 39.2 | 100 |
| *ZNF264* | 57702922 | 0.713 | 0.006 | 59.5 | 83.3 |
| *ZNF264* | 57702918 | 0.709 | 0.020 | 50.2 | 87.5 |
| *ZNF264* | 57702865 | 0.638 | 0.033 | 33.5 | 95.8 |
| *ZNF264* | 57702861 | 0.742 | 0.020 | 55.5 | 91.7 |
| *ZNF264* | 57702856 | 0.679 | 0.041 | 38.3 | 95.8 |
| *ZNF264* | 57702847 | 0.612 | 0.002 | 48.9 | 70.8 |
| *ZNF264* | 57702838 | 0.659 | 0.016 | 37.9 | 91.7 |
| *ZNF264* | 57702829 | 0.718 | 0.019 | 52.9 | 87.5 |
| *ZNF264* | 57702826 | 0.687 | 0.020 | 45.8 | 91.7 |
| *ZNF264* | 57702819 | 0.742 | 0.006 | 63.0 | 79.2 |
| *ZNF264* | 57702816 | 0.714 | 0.072 | 39.2 | 100 |
| *ZNF264* | 57702810 | 0.444 | 0.002 | 47.1 | 62.5 |
| *ZNF264* | 57702804 | 0.722 | 0.067 | 38.3 | 100 |
| *ZNF264* | 57702794* | 0.744 | 0.006 | 66.1 | 79.2 |
| *ZNF264* | 57702791 | 0.730 | 0.005 | 64.3 | 75.0 |
| *ZNF264* | 57702776 | 0.731 | 0.025 | 50.2 | 91.7 |
| *ZNF264* | 57702773 | 0.743 | 0.024 | 59.0 | 83.3 |
| *ZNF264* | 57702769 | 0.739 | 0.028 | 53.7 | 95.8 |
| *ZNF264* | 57703278 | 0.745 | 0.016 | 58.1 | 91.7 |
| *ZNF264* | 57703286 | 0.763 | 0.022 | 60.4 | 100 |
| *ZNF264* | 57703292 | 0.765 | 0.023 | 59.5 | 100 |
| *ZNF264* | 57703301^†^ | 0.788 | 0.023 | 56.4 | 100 |
| *ZNF264* | 57703306 | 0.790 | 0.021 | 61.2 | 100 |
| *ZNF264* | 57703324 | 0.789 | 0.036 | 61.7 | 100 |
| *ZNF264* | 57703333 | 0.780 | 0.063 | 60.4 | 100 |
| *ZNF264* | 57703335 | 0.754 | 0.045 | 59.5 | 100 |
| *ZNF264* | 57703342 | 0.806 | 0.062 | 60.4 | 100 |
| *ZNF264* | 57703356 | 0.790 | 0.040 | 67.8 | 95.8 |
| *ZNF264* | 57703364 | 0.779 | 0.081 | 60.4 | 100 |
| *ZNF264* | 57703371 | 0.770 | 0.058 | 64.8 | 91.7 |
| *ZNF264* | 57703411 | 0.793 | 0.158 | 65.2 | 91.7 |
| *ZNF264* | 57703417 | 0.804 | 0.113 | 64.3 | 95.8 |
| *ZNF264* | 57703420 | 0.794 | 0.120 | 63.0 | 95.8 |
| *ZNF264* | 57703424 | 0.784 | 0.196 | 63.4 | 91.7 |
| *ZNF264* | 57703469 | 0.798 | 0.153 | 63.4 | 95.8 |

Note: * The target CpG of *ZNF264* (cg26970847) in the annotation information of Infinium 450K array, and ^†^ The target CpG of *ZNF264* (cg11140785) in the annotation information of Infinium 450K array.

# Table S14. Receiver operating characteristic curve analyses of 16 CpG sites in target region of *ZNF543* between colorectal cancer and adjacent normal tissues in our inhouse primary validation dataset.

| **Gene** | **Genomic Coordinate** | **AUC** | **Cut-off Value** | **Sensitivity (%)** | **Specificity (%)** |
| --- | --- | --- | --- | --- | --- |
| *ZNF543* | 57831754 | 0.705 | 0.05 | 52.9 | 100 |
| *ZNF543* | 57831757 | 0.746 | 0.052 | 53.3 | 100 |
| *ZNF543* | 57831761 | 0.744 | 0.052 | 52.9 | 100 |
| *ZNF543* | 57831763 | 0.718 | 0.048 | 53.3 | 100 |
| *ZNF543* | 57831781 | 0.738 | 0.049 | 51.5 | 100 |
| *ZNF543* | 57831793 | 0.721 | 0.022 | 55.1 | 95.8 |
| *ZNF543* | 57831797 | 0.720 | 0.058 | 51.1 | 100 |
| *ZNF543* | 57831806 | 0.719 | 0.062 | 52.4 | 100 |
| *ZNF543* | 57831816 * | 0.699 | 0.06 | 52.9 | 100 |
| *ZNF543* | 57831819 | 0.736 | 0.052 | 52.0 | 100 |
| *ZNF543* | 57831834 | 0.748 | 0.019 | 55.9 | 95.8 |
| *ZNF543* | 57831839 | 0.747 | 0.020 | 55.1 | 95.8 |
| *ZNF543* | 57831842 | 0.736 | 0.022 | 55.5 | 95.8 |
| *ZNF543* | 57831844 | 0.737 | 0.013 | 56.8 | 95.8 |
| *ZNF543* | 57831849 | 0.74 | 0.019 | 53.7 | 100 |
| *ZNF543* | 57831858 | 0.739 | 0.018 | 55.5 | 100 |

Note: * The target CpG of *ZNF543* (cg14786398) in the annotation information of Infinium 450K array.

# Table S15. The demographic and clinical characteristics of the CRC, precancerous lesions, and healthy controls in the cfDNA validation cohort.

| **Characteristics** | **CRC** | **Healthy controls** | **Adenoma** | **Polyp** |
| --- | --- | --- | --- | --- |
| Total (n) | 195 | 103 | 22 | 6 |
| Gender, n (%) * |  |  |  |  |
| Male | 112 (57.4%) | 48 (46.6%) | 13 (59.1%) | 1 (83.3%) |
| Female | 83 (42.6%) | 55 (53.4%) | 9 (40.9%) | 5 (16.7%) |
| Age (years)* |  |  |  |  |
| Mean ± SD | 60.3±10.4 | 51.3±12.2 | 59.4±8.8 | 51.7±9.0 |
| Range | 34-85 | 25-74 | 37-74 | 43-67 |
| Localization, n (%) |  | - | - | - |
| Colon | 94 (48.2%) |  |  |  |
| Rectum | 101 (51.8%) |  |  |  |
| AJCC stage, n (%) |  | - | - | - |
| 0 | 6 (3.1%) |  |  |  |
| Ⅰ | 33 (16.9%) |  |  |  |
| Ⅱ | 65 (33.3%) |  |  |  |
| Ⅲ | 56 (28.7%) |  |  |  |
| Ⅳ | 18 (9.2%) |  |  |  |
| Not reported | 17 (8.7%) |  |  |  |
| CEA, n (%) |  | - | - | - |
| ≥ 5 ng/mL | 86 (44.1%) |  |  |  |
| < 5 ng/mL | 109 (55.9%) |  |  |  |
| Not reported | 0 |  |  |  |

Note: There was no statistical difference in gender distribution between CRC and healthy controls (*P* = 0.08), and statistically significant differences (*P* < 0.05) was found between age. CRC: Colorectal cancer; SD: Standard deviation; AJCC: American Joint Commission on Cancer; CEA: carcinoembryonic antigen.

# Table S16. The demographic and clinical characteristics of the CRC and healthy controls in the age-matched cfDNA validation cohort.

| **Characteristics** | **CRC** | **Healthy controls** |
| --- | --- | --- |
| Total (n) | 92 | 92 |
| Gender, n (%) * |  |  |
| Male | 45 (48.9%) | 44 (47.8%) |
| Female | 47 (51.1%) | 48 (52.2%) |
| Age (years) |  |  |
| Mean ± SD | 54.7±9.3 | 54.1±9.7 |
| Range | 34-74 | 30-74 |
| Localization, n (%) |  | - |
| Colon | 50 (54.3%) |  |
| Rectum | 42 (45.7%) |  |
| AJCC stage, n (%) |  | - |
| 0 | 2 (2.2%) |  |
| Ⅰ | 13 (14.1%) |  |
| Ⅱ | 37 (40.2%) |  |
| Ⅲ | 29 (31.5%) |  |
| Ⅳ | 7 (7.6%) |  |
| Not reported | 4 (4.3%) |  |
| CEA, n (%) |  | - |
| ≥ 5 ng/mL | 38 (41.3%) |  |
| < 5 ng/mL | 54 (58.7%) |  |
| Not reported | 0 |  |

Note: There was no statistical difference in gender distribution between CRC and healthy controls (*P* > 0.05). CRC: Colorectal cancer; SD: Standard deviation; AJCC: American Joint Commission on Cancer; CEA: carcinoembryonic antigen.


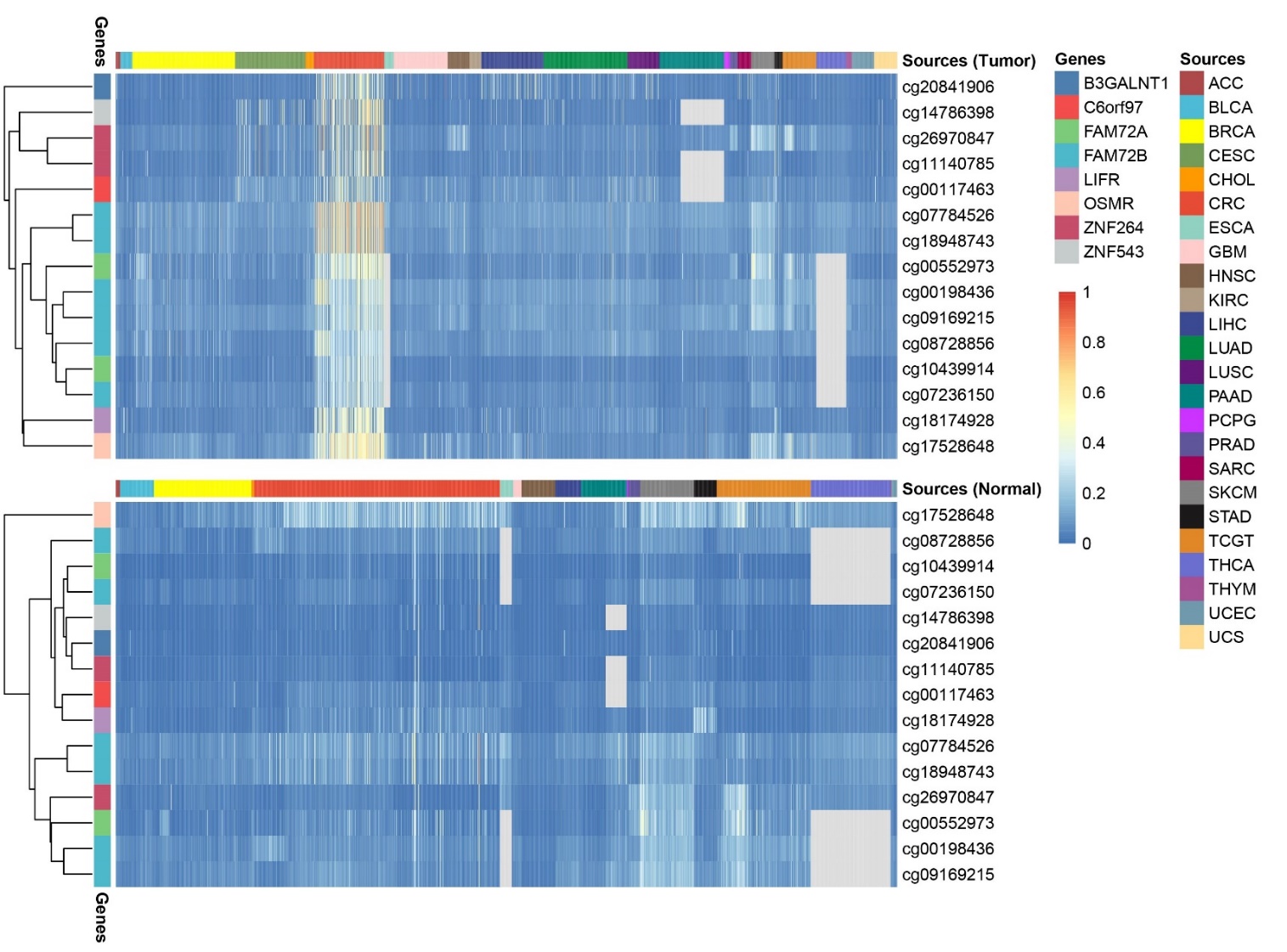


# Figure S1. Validation of 15 CRC-specific methylation CpG sites in multiple types of cancer tissues and normal tissues from GEO database.

Note: Unsupervised hierarchical clustering of 15 CRC-specific DNA methylation markers in 3,000 tumor and 1,061 normal tissues from 24 types of cancer. The gray area represents a missing of methylation levels at this CpG site.

**
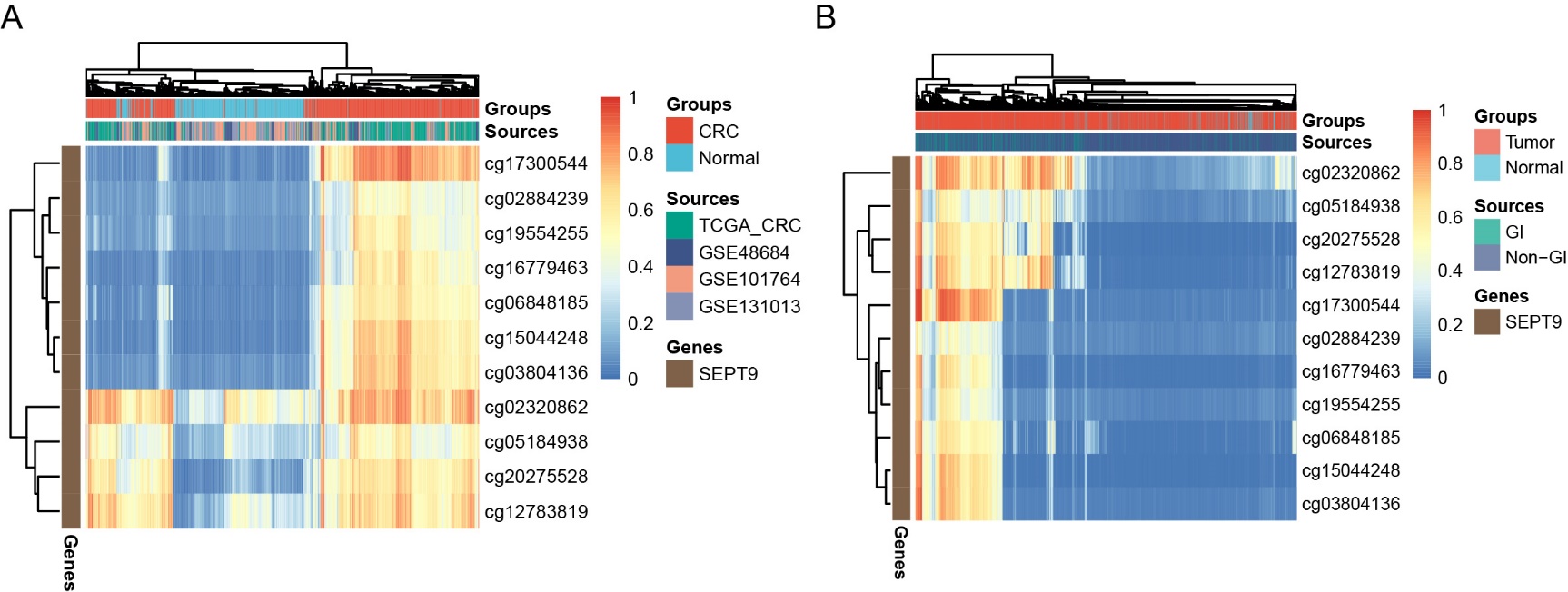
**

# Figure S2. Methylation levels of 11 CpG sites in the *SEPT9* promoter in 29 types of cancer tissues from TCGA database.

Note: (A) Unsupervised hierarchical clustering of the 11 CpG sites in 395 CRC samples and 45 matched adjacent normal tissue samples. (B) Unsupervised hierarchical clustering of 11 CpG sites in 7925 tumor and 704 normal tissues of 28 other cancer types. The GI-related tumors mainly include esophageal carcinoma, hepatocellular carcinoma, pancreatic adenocarcinoma and stomach adenocarcinoma.


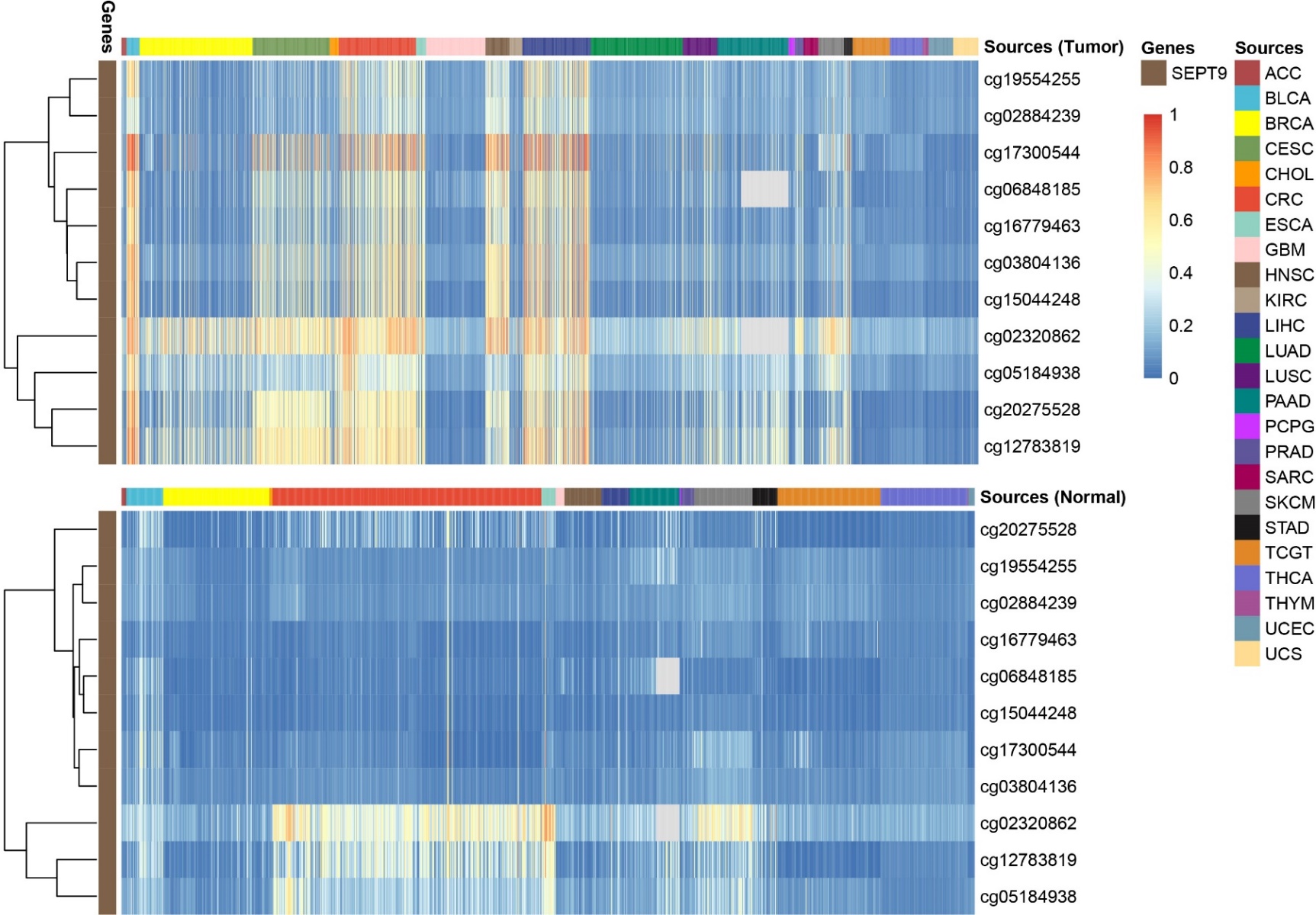


# Figure S3. Validation of 11 CpG sites in the *SEPT9* promoter in multiple types of cancer tissues and normal tissues from GEO database.

Note: Unsupervised hierarchical clustering of 11 CpG sites of *SEPT9* promoter in 3,000 tumor and 1,061 normal tissues from 24 types of cancer.


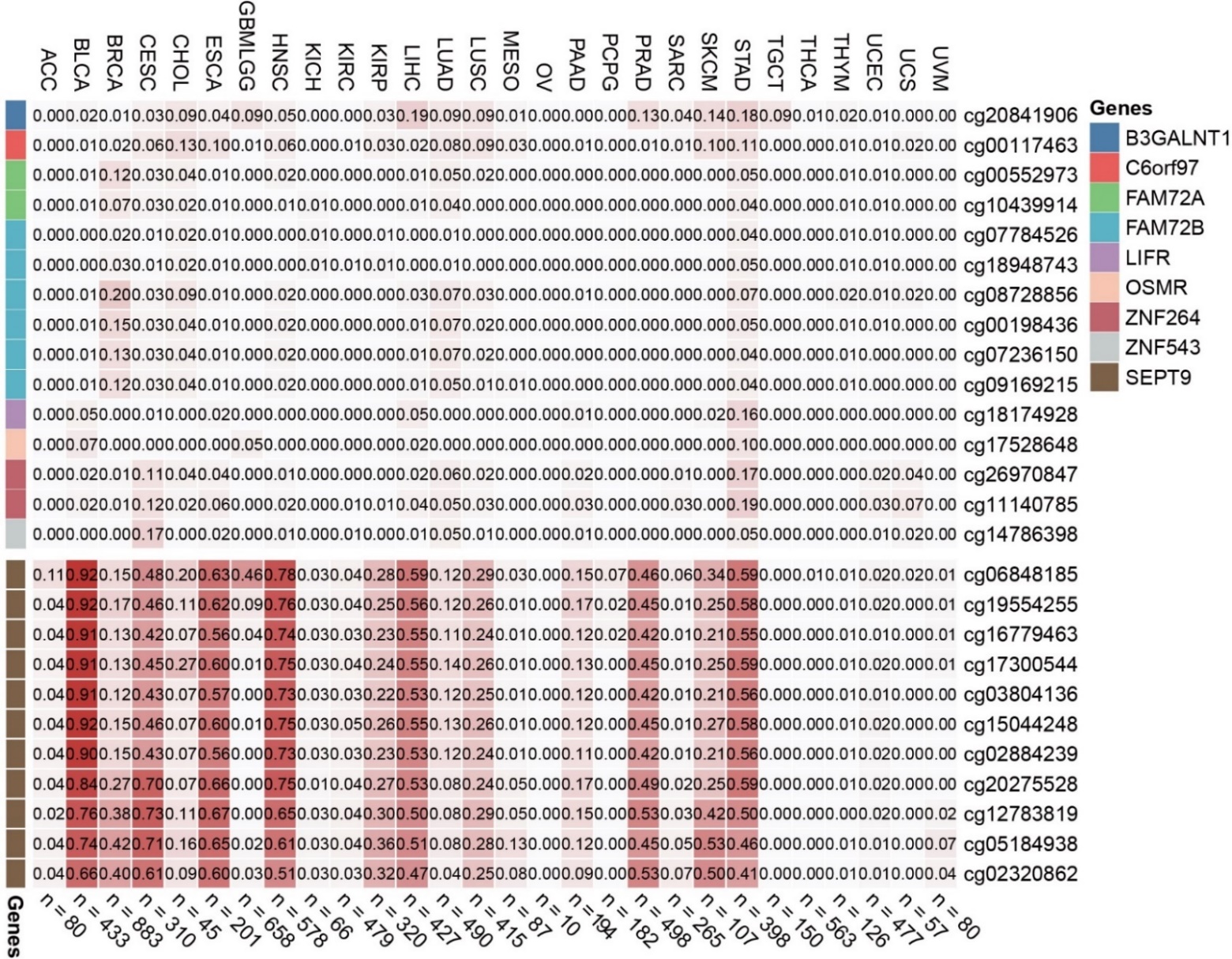


# Figure S4. Heatmap for misclassification rate of our 15 CpG sites and 11 CpG sites of *SPET9* for distinguishing CRC samples from 28 other cancer types in TCGA dataset.

Note: n represents the number of samples of each type of tumor and normal tissue.


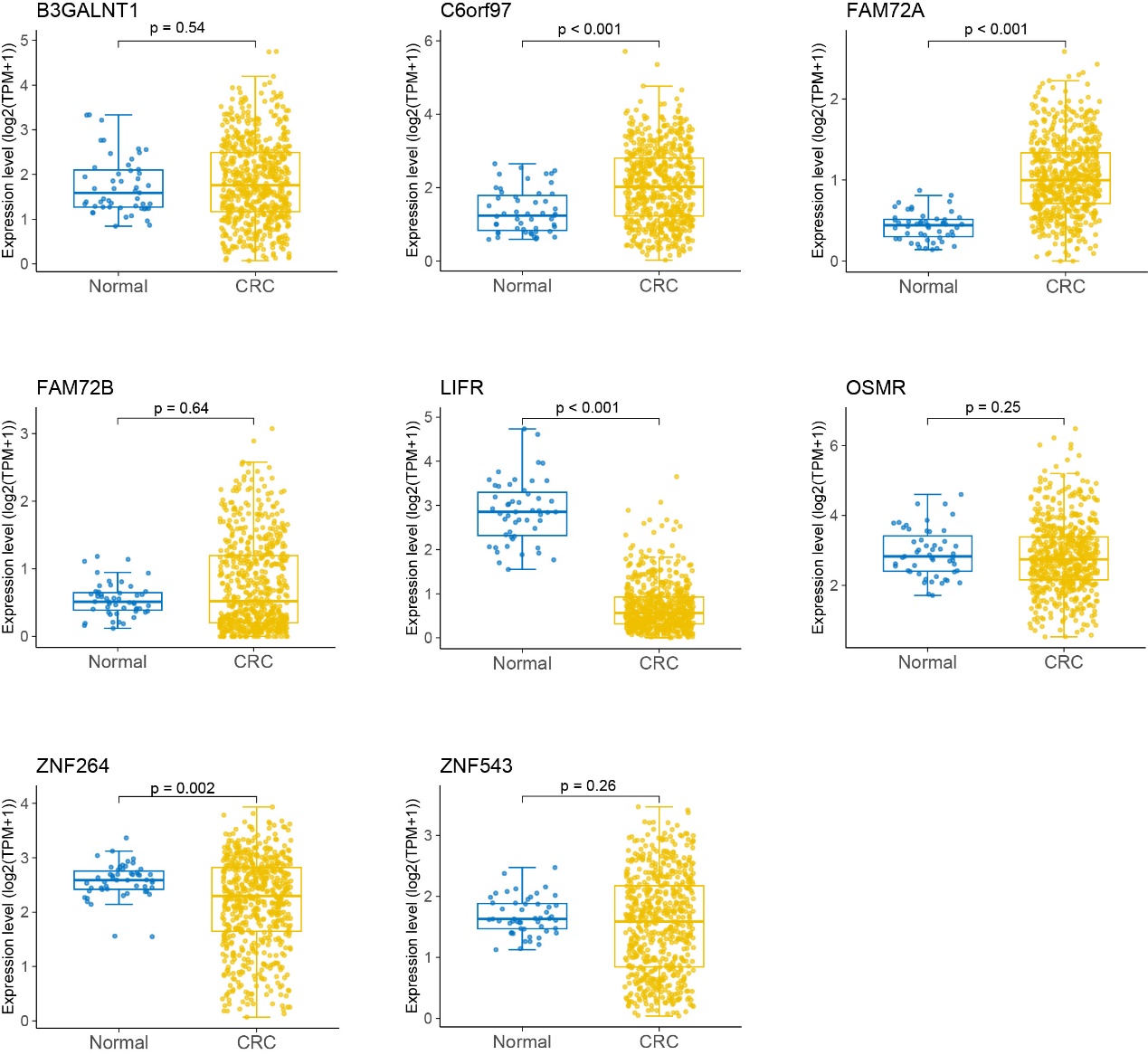


# Figure S5. Boxplots for eight gene expression in CRC tissues and adjacent normal tissues.


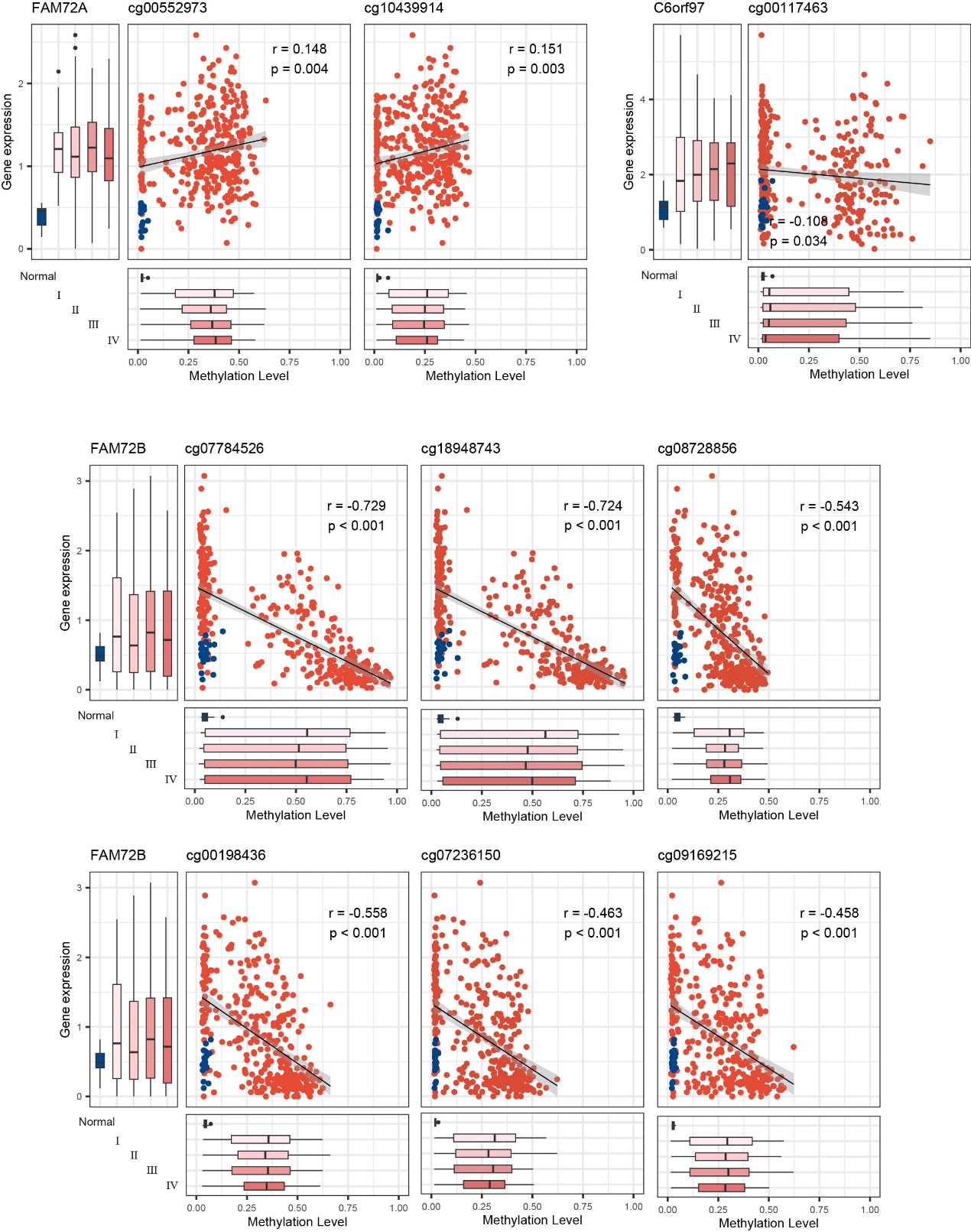


# Figure S6. Spearman correlation analysis between methylation levels of nine CpG sites and gene expression of *FAM72A*, *C6orf97*, and *FAM72B* in the TCGA CRC dataset.

Note: r, Spearman’s correlation coefficient. Left boxplot represents the expression level of the gene in CRC tissues in different tumor stages and adjacent normal tissue. The boxplot below represents the methylation level of the CpG in CRC tissues in different tumor stages and adjacent normal tissue.


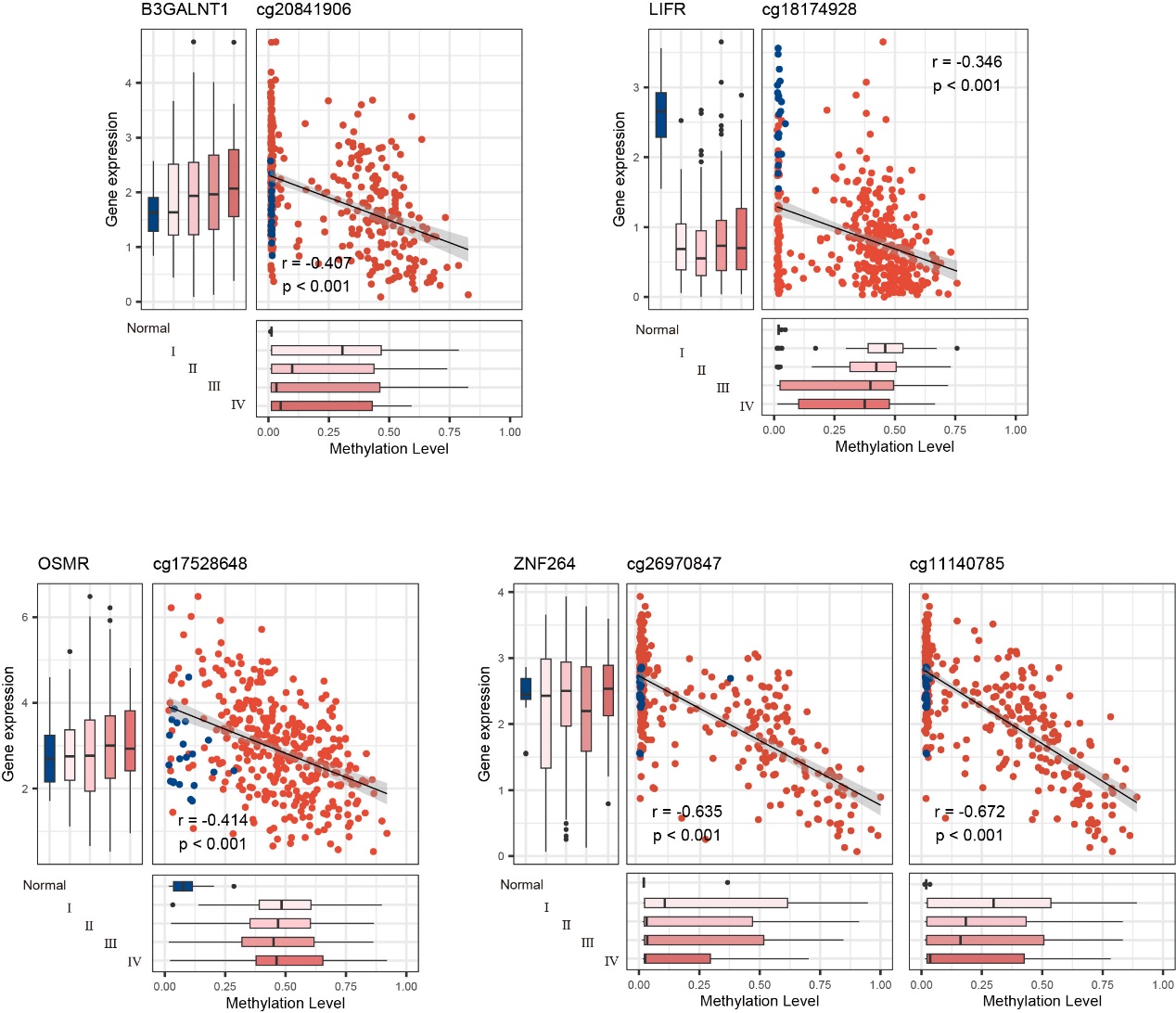


# Figure S7. Spearman correlation analysis between methylation levels of six CpG sites and gene expression of *B3GALNT1*, *LIFR*, *OSMR* and *ZNF264* in the TCGA CRC dataset.

Note: r, Spearman’s correlation coefficient. Left boxplot represents the expression level of the gene in CRC tissues in different tumor stages and adjacent normal tissue. The boxplot below represents the methylation level of the CpG in CRC tissues in different tumor stages and adjacent normal tissue.


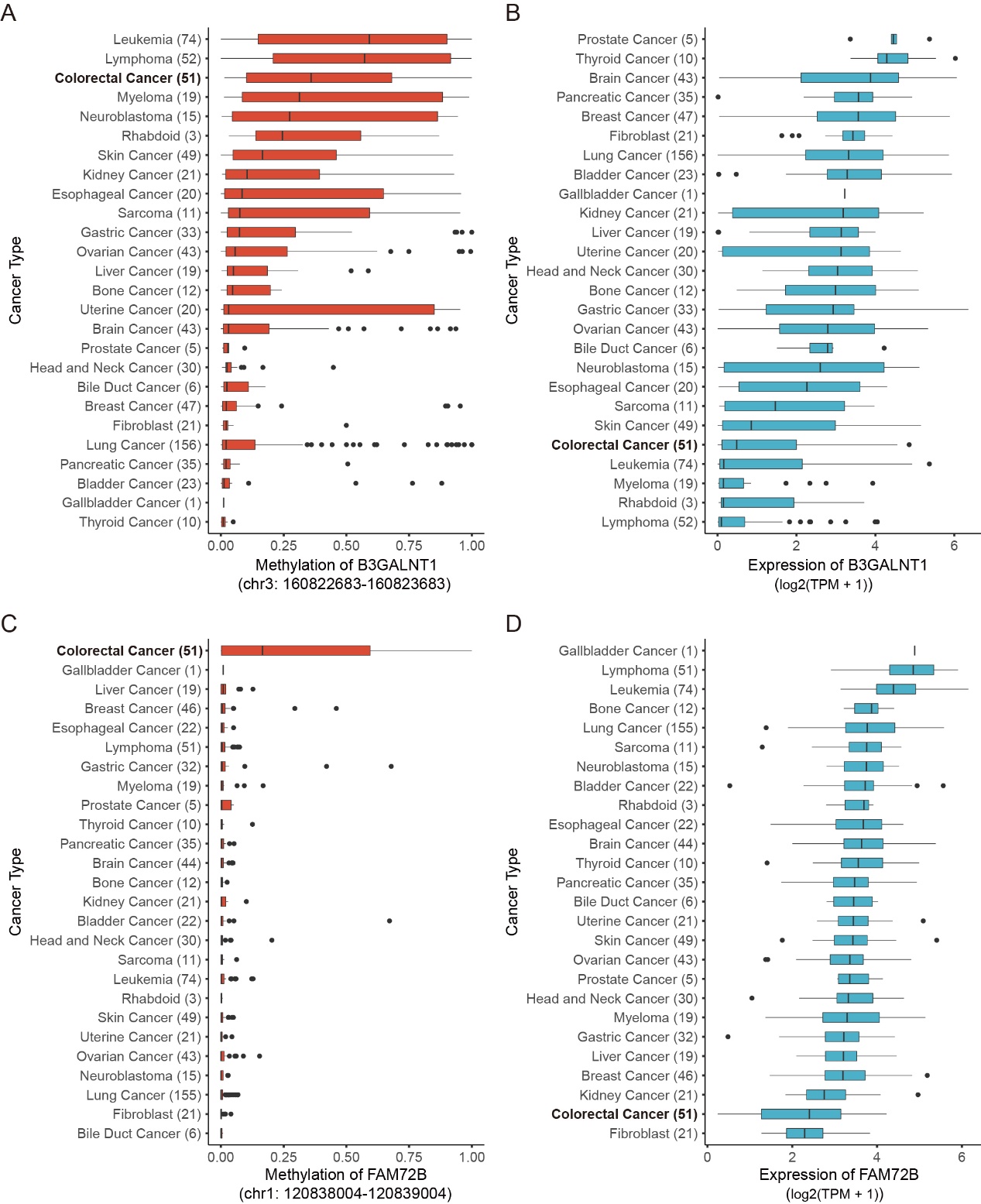


# Figure S8. Methylation (RRBS) level (A) and expression level (B) of *B3GALNT1* gene in multiple types of cancer cell lines from Cancer Cell Line Encyclopedia. (C) Methylation (RRBS) of *FAM72B*. (D) mRNA expression (RNA-seq) of *FAM72B*.

Note: The methylation level is the average methylation level of all CpG sites within 1,000 bp. The methylated region of *B3GALNT1* contains the target CpG site cg20841906 (chr3: 160822911). The methylated region of *FAM72B* includes target CpG sites cg07784526 (chr1: 120838320), cg18948743 (chr1: 120838323), and cg08728856 (chr1: 120838718).


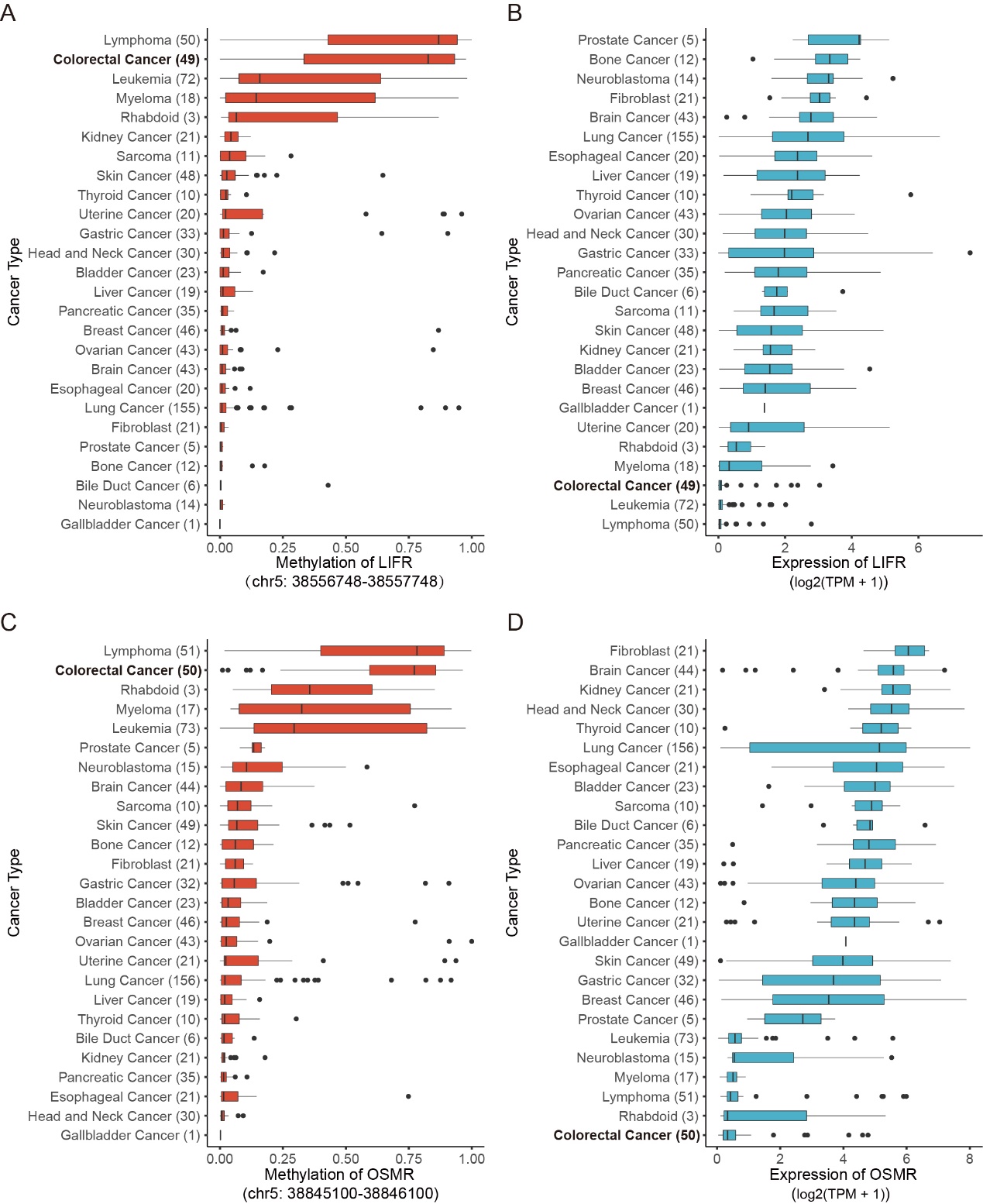


# Figure S9. Methylation (RRBS) level (A) and expression level (B) of *LIFR* gene in multiple types of cancer cell lines from Cancer Cell Line Encyclopedia. (C) Methylation (RRBS) of *OSMR*. (D) mRNA expression (RNA-seq) of *OSMR*.

Note: The methylation level is the average methylation level of all CpG sites within 1,000 bp. The methylated region of *LIFR* contains the target CpG site cg18174928 (chr5: 38557085). The methylated region of *OSMR* contains the target CpG site cg17528648 (chr5: 38846100).


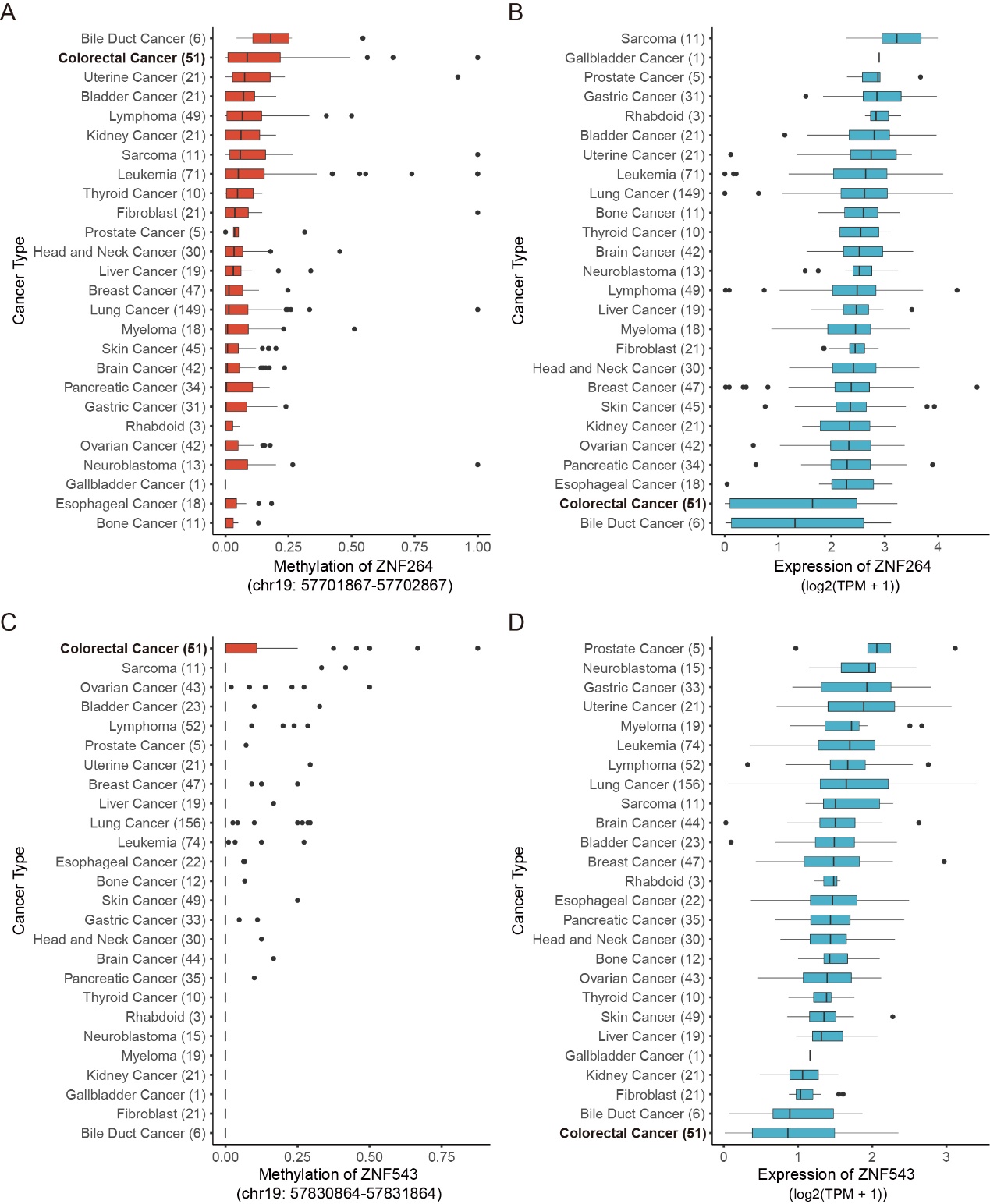


# Figure S10. Methylation (RRBS) level (A) and expression level (B) of *ZNF264* gene in multiple types of cancer cell lines from Cancer Cell Line Encyclopedia. (C) Methylation (RRBS) of *ZNF543*. (D) mRNA expression (RNA-seq) of *ZNF543*.

Note: The methylation level is the average methylation level of all CpG sites within 1,000 bp. The methylated region of *ZNF264* contains the target CpG site cg26970847 (chr19: 57702793). The methylated region of *ZNF543* contains the target CpG site cg14786398 (chr19: 57831816).


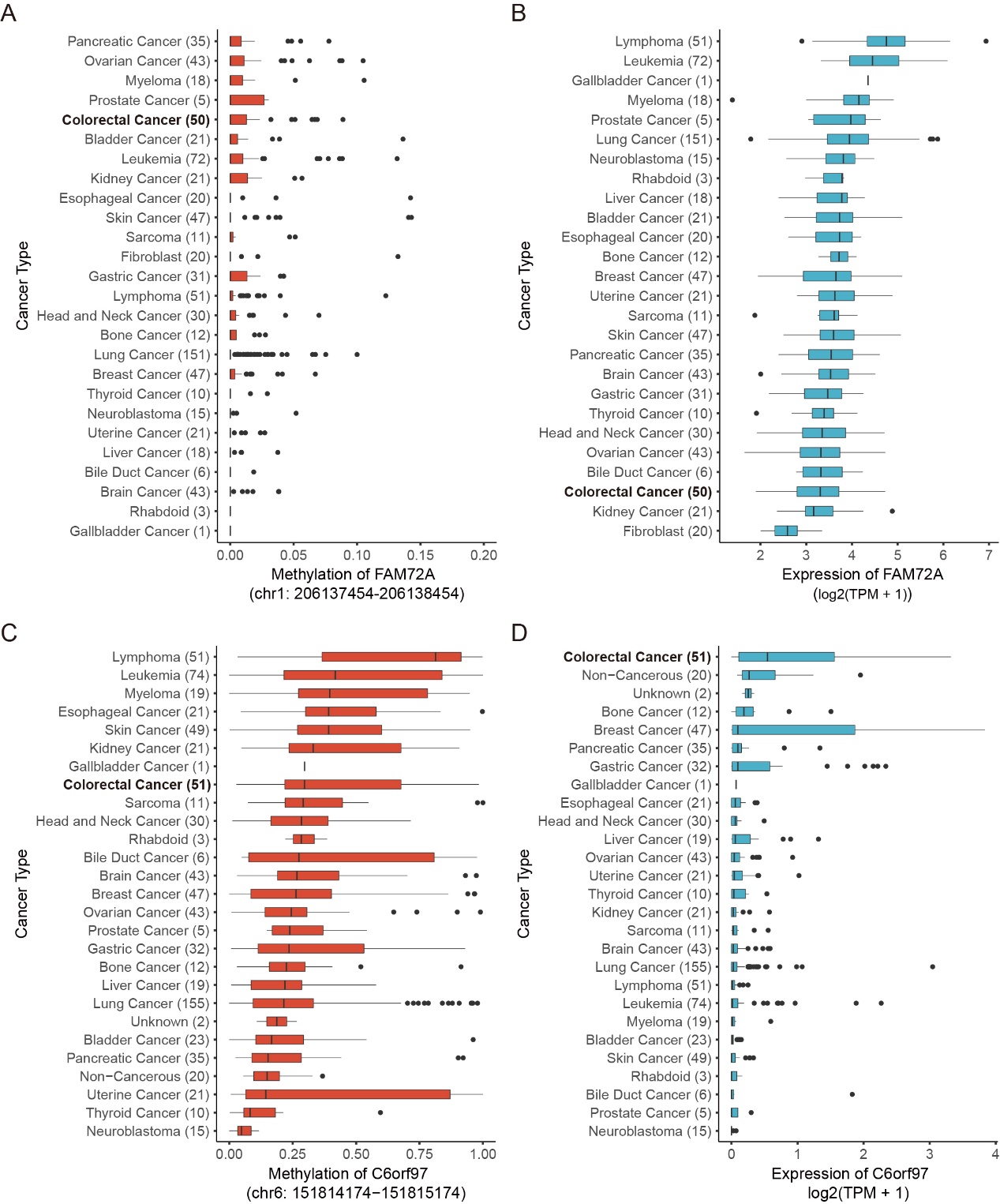


# Figure S11. Methylation (RRBS) level (A) and expression level (B) of *FAM72A* gene in multiple types of cancer cell lines from Cancer Cell Line Encyclopedia. (C) Methylation (RRBS) of *C6orf97*. (D) mRNA expression (RNA-seq) of *C6orf97*.

Note: The methylation level is the average methylation level of all CpG sites within 1,000 bp. The methylated region of *FAM72A* contains the target CpG site cg10439914 (chr19: 206137759). The methylated region of *C6orf97* does not contain the target CpG site


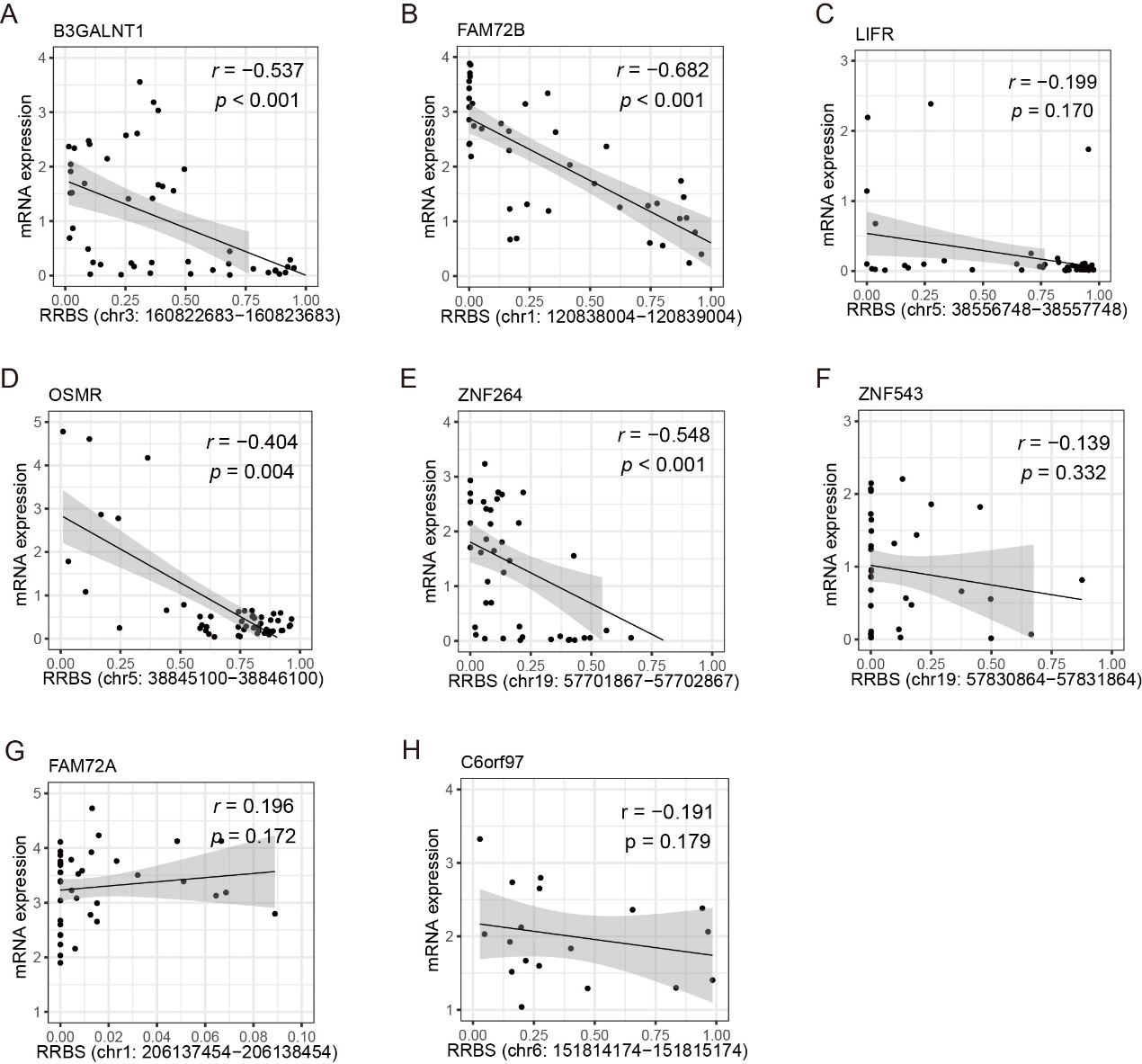


# Figure S12. Correlation between methylation and expression levels of (A) *B3GALNT1*, (B) *FAM72B*, (C) *LIFR*, (D) *OSMR*, (E) *ZNF264*, (F) *ZNF543*, (G) *FAM72A* and (H) *C6orf97* in CRC cell lines.


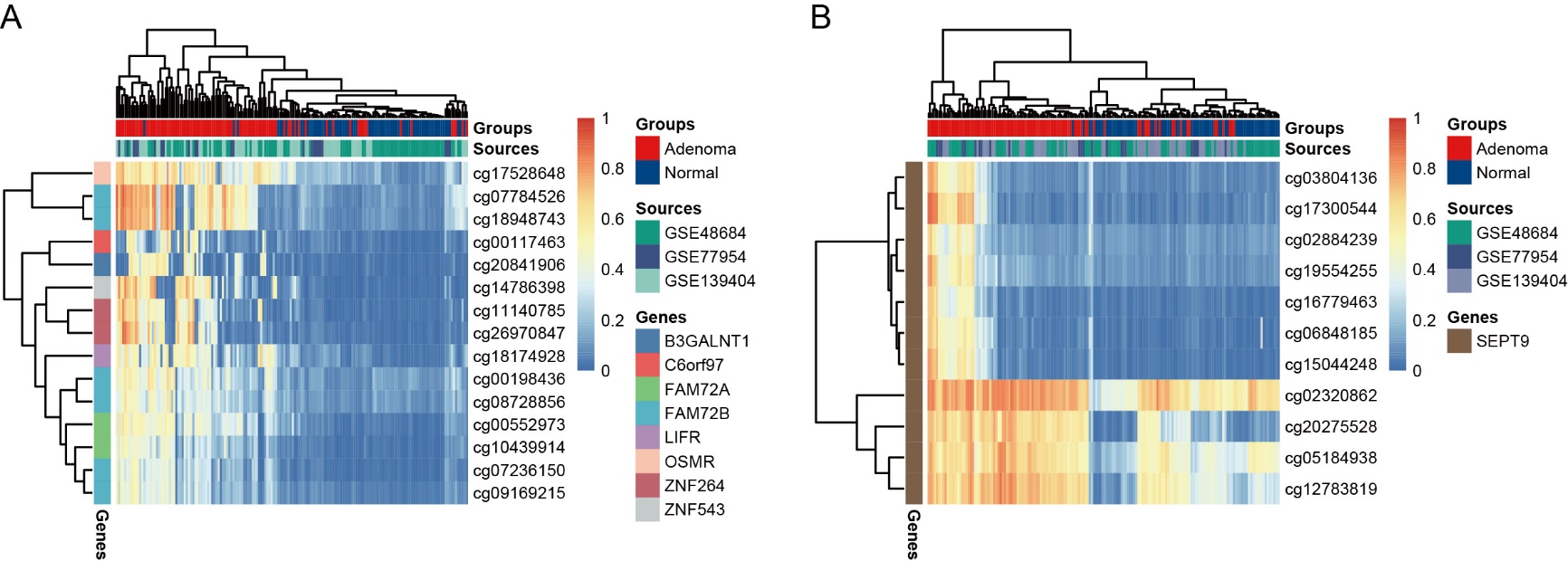


# Figure S13. Validation of 15 CRC-specific methylation CpG sites of our study (A) and 11 CpG sites in the *SEPT9* promoter (B) in tissues of colorectal adenomas and normal tissue samples.

Note: Unsupervised hierarchical clustering of these CpG sites in 94 colorectal adenomas and 72 normal tissue samples.


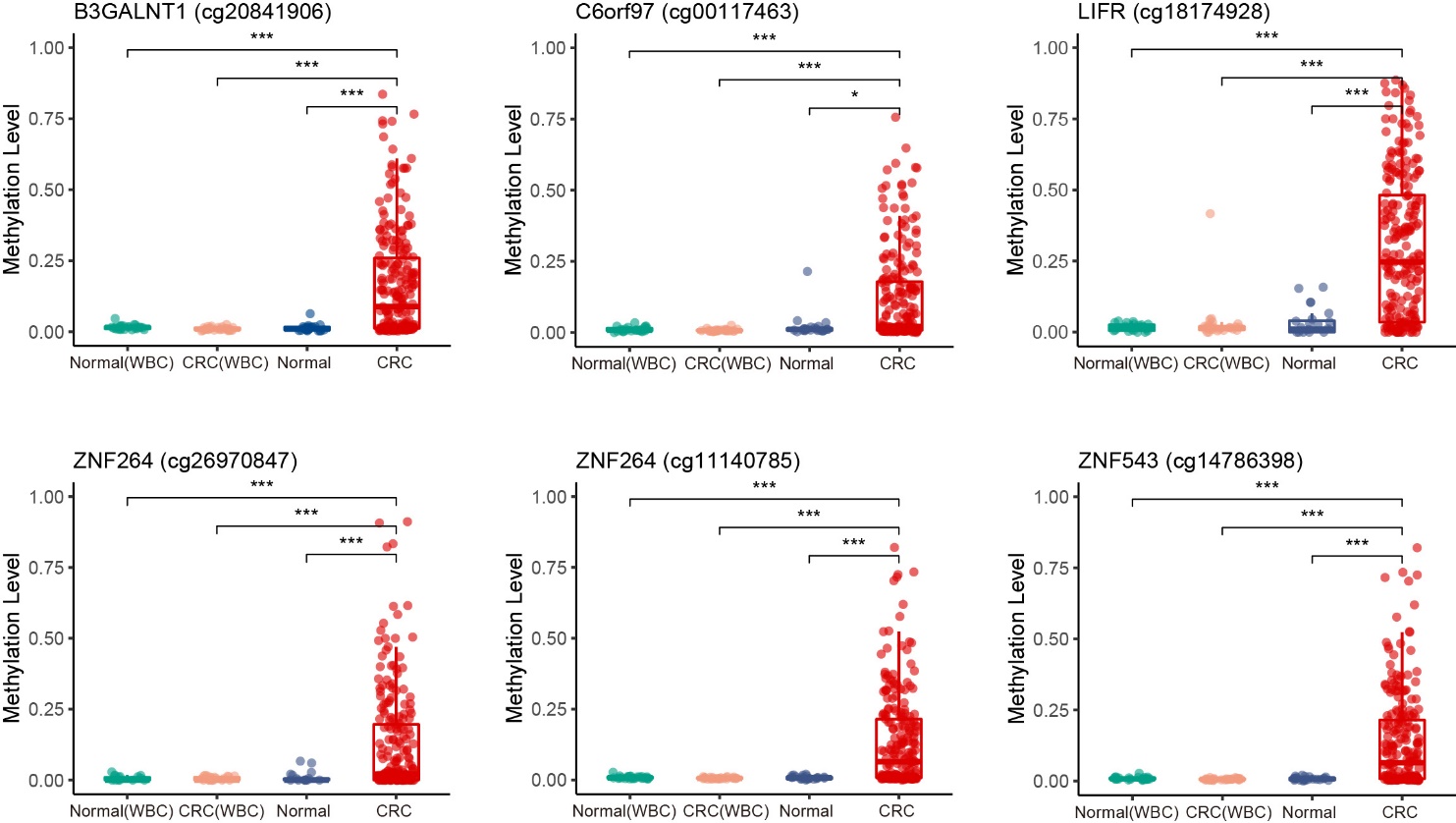


# Figure S14. The methylation level of the target region in the validation cohort.

Note: Boxplot and dot-plot of methylation levels of candidate CpG sites of *B3GALNT1*, *C6orf97*, *LIFR*, *ZNF264*, and *ZNF543* in CRC tissues, normal tissues, and WBC samples from CRC patients and healthy controls. Symbols indicate statistical significance: ***, *P* ≤ 0.001.


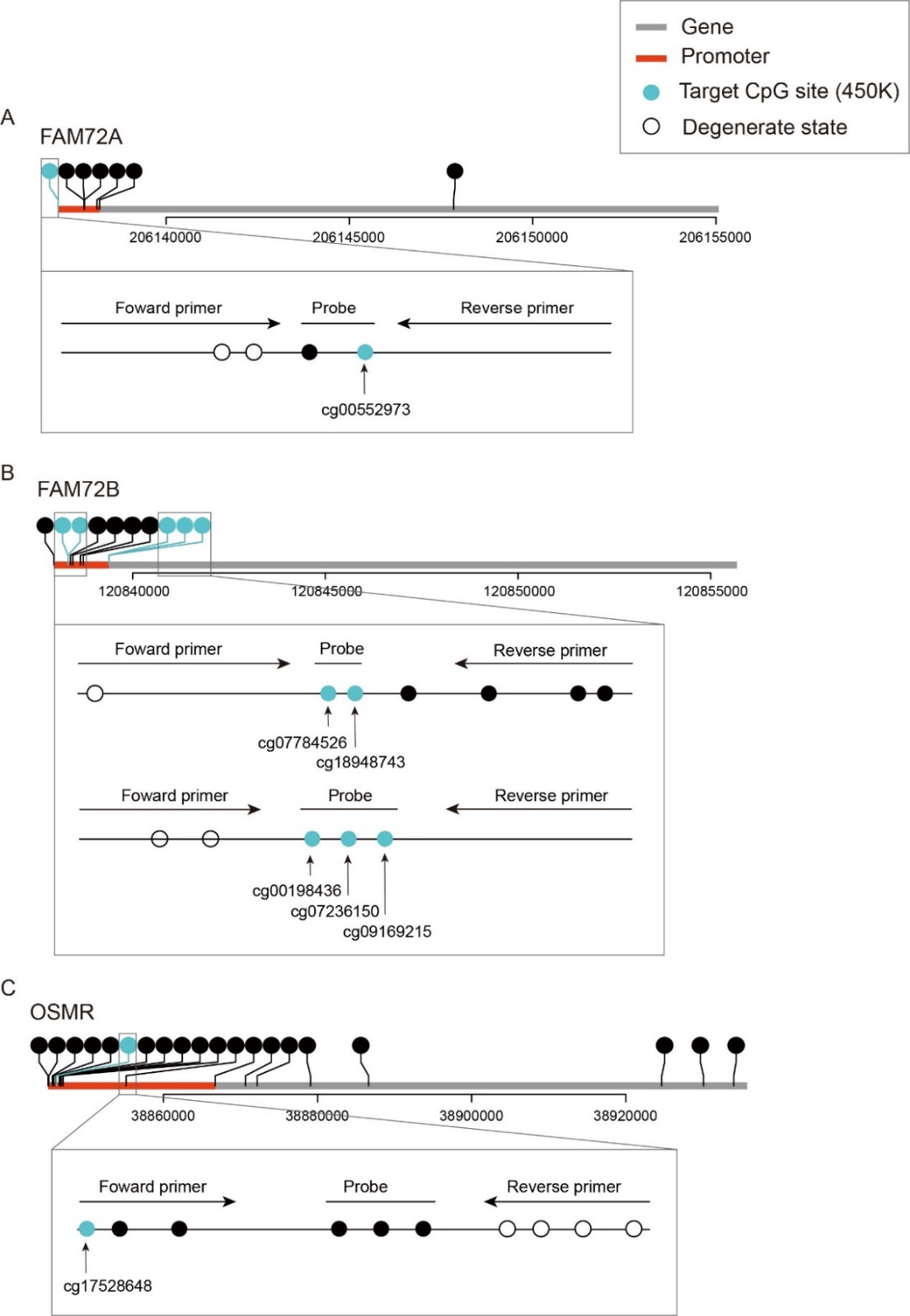


# Figure S15. Schematic illustration of the localization of target CpG sites (Infinium 450K) and droplet digital PCR assays related to the presence of CpG sites in the genomic region of *FAM72A* (A), *FAM72B* (B), and *OSMR* (C).


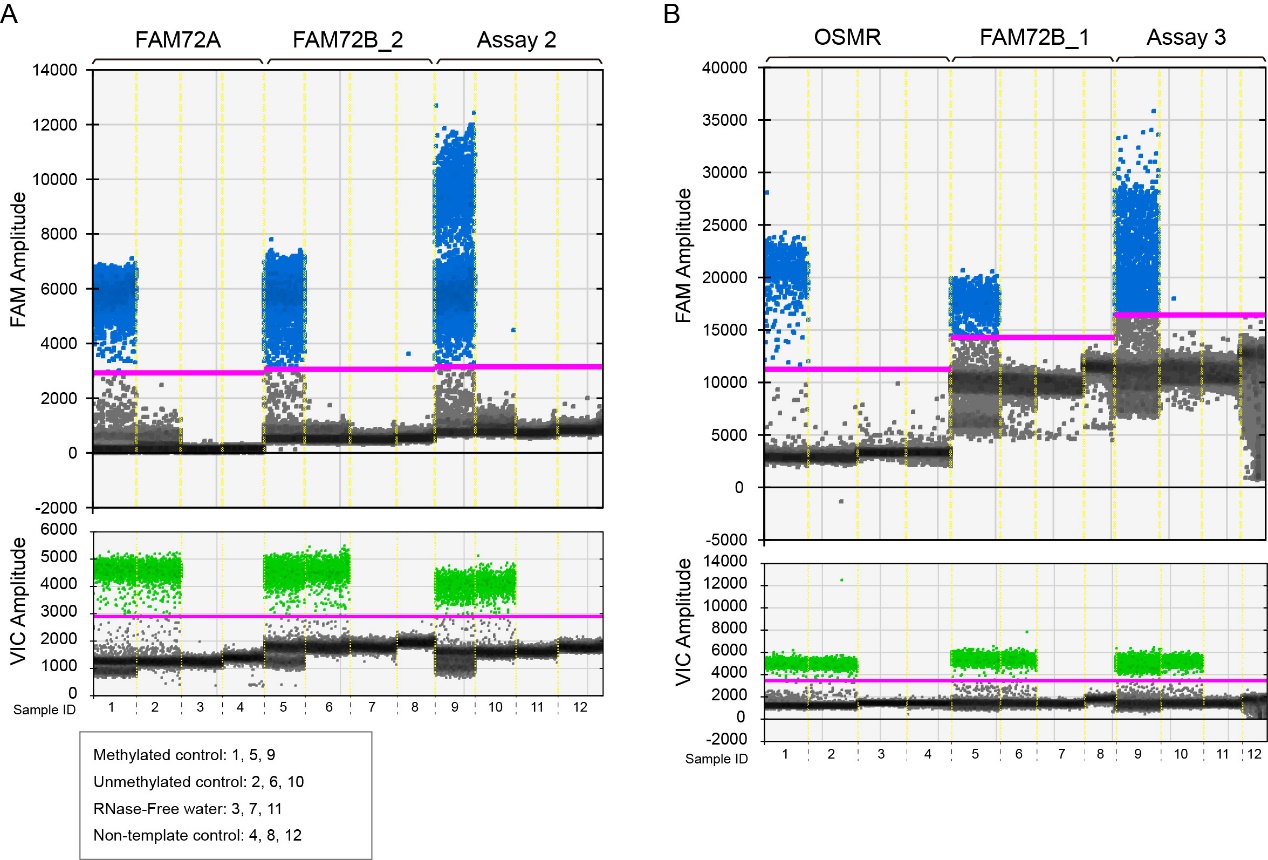


# Figure S16. Combination and construction of multiplex droplet digital PCR assays. (A) Assay 2, and (B) Assay 3.

Note: Each single amplification system or multiplex amplification system amplifies four templates including methylated control, unmethylated control, RNase-free water, and non-template control. The solid pink line is the manually set threshold for dividing positive and negative droplets.

**
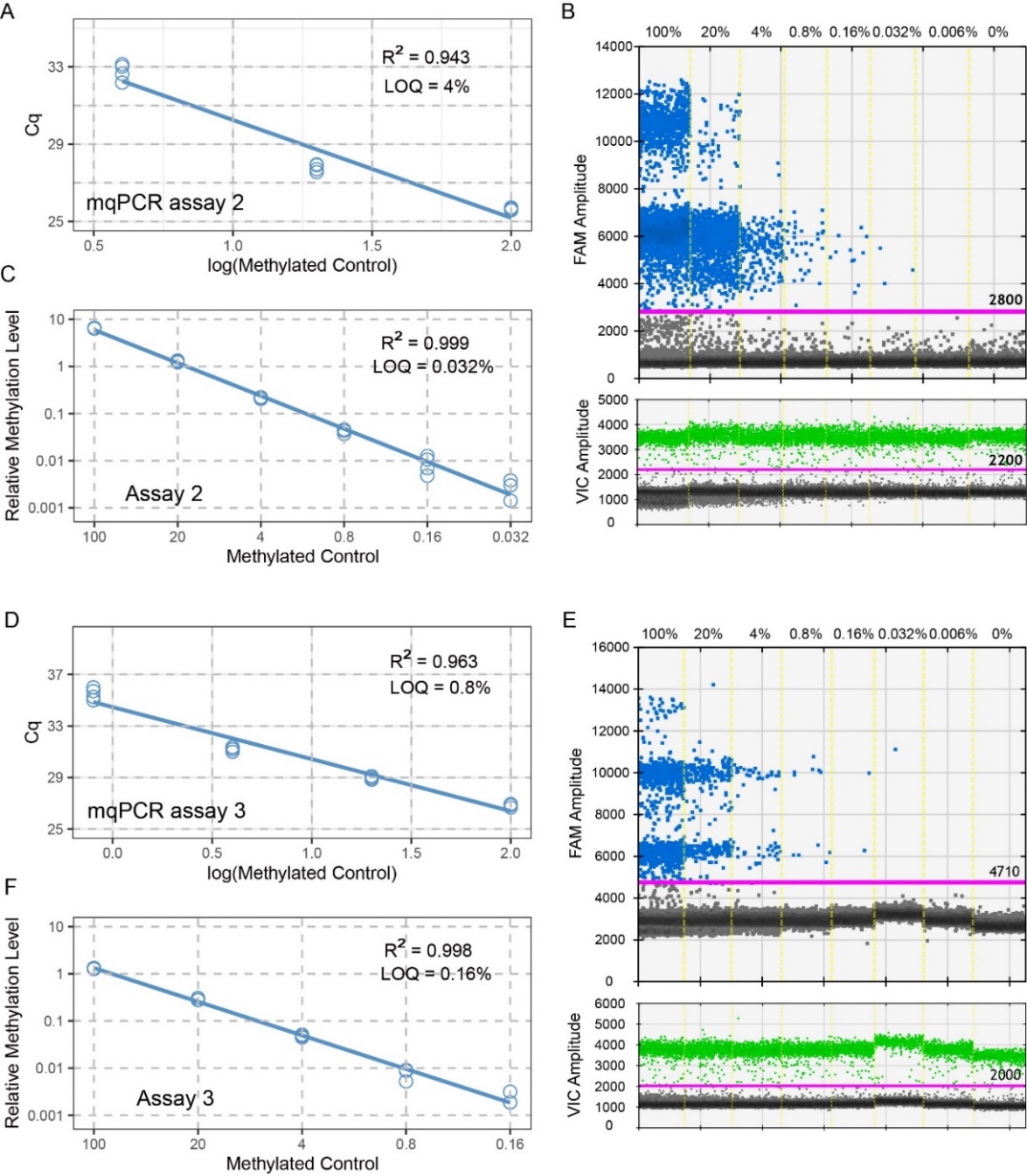
**

# Figure S17. Comparative analysis of limit of quantification (LOQ) for multiplex droplet digital PCR (mddPCR) and conventional multiplex MethyLight PCR (mqPCR).

Note: (A) Standard curve of quantification between Cq value and log transformation of serially diluted methylated controls in mqPCR assay 2. (B) Quantasoft amplification plots of 5-fold dilution series of methylated controls in Assay 2. The solid pink line is manually set threshold, which is used for dividing positive and negative droplets. (C) Standard curve of quantification between relative methylation level and serially diluted methylated controls in Assay 2. The amplification performance of Assay 3 and mqPCR assay 3 is shown in (D), (E), and (F). All DNA controls were run in four replicates. The X-axis displays the concentration (or log- transformed) of methylated controls and the Y-axis represents the values of quantitative cycle (Cq) or relative methylation level (The ratio of methylated molecules copies of target region and reference gene (ACTB) of each methylated control).


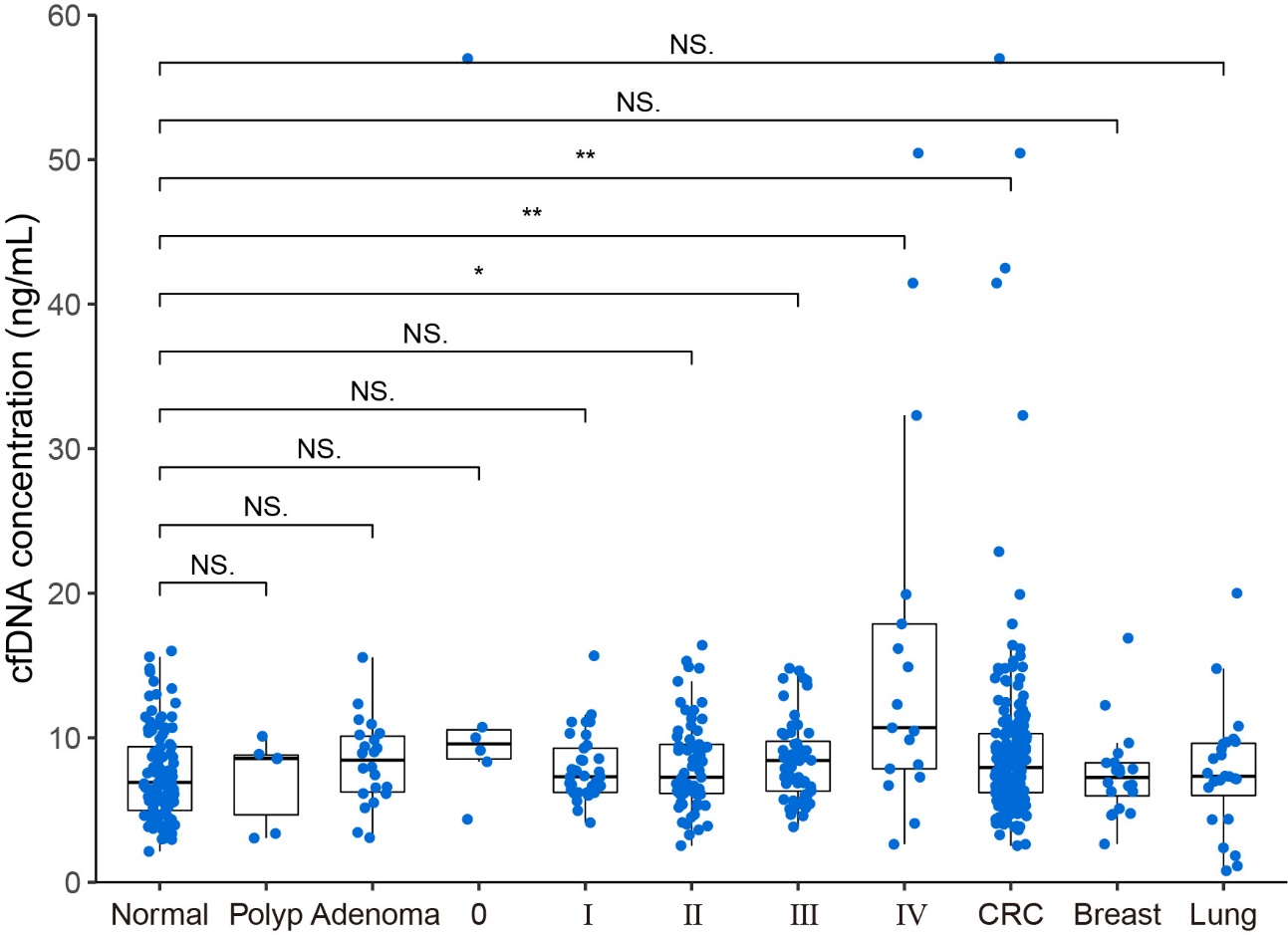


# Figure S18. Analysis of cfDNA extraction concentrations in our cfDNA validation cohort.

Note: A total of 370 cfDNA extraction samples (103 Healthy controls, 6 polyps, 22 adenomas, 6 CRC stage 0, 33 CRC stage I, 65 CRC stage II, 56 CRC stage III, 18 CRC stage IV, 195 all CRC patients (including 17 without CRC stage), 20 breast diseases, and 24 lung diseases) were measured and compared for the cfDNA concentration. Symbols indicating statistical significance: NS, *P* > 0.05; *, *P* ≤ 0.05; **, *P* ≤ 0.01.


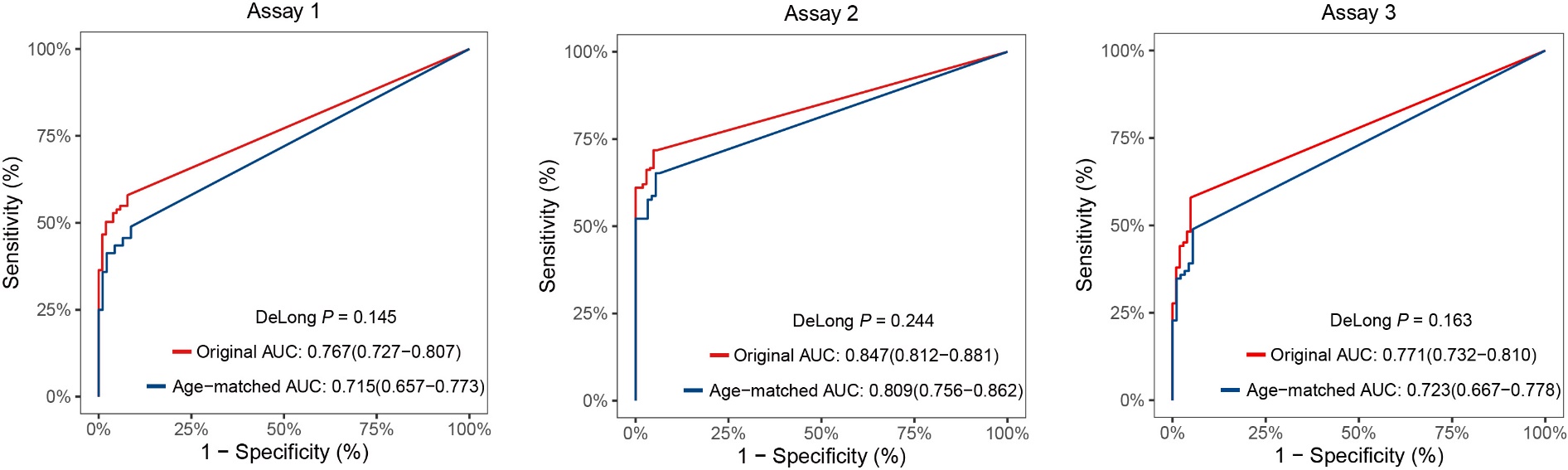


# Figure S19. Receiver operating characteristics curves of Assay 1, Assay 2, and Assay 3 for distinguishing colorectal cancer from healthy controls in original or age-matched cfDNA cohort.

Note: The AUC values between groups were compared using the DeLong method.
